# Mutations responsible for the carbapenemase activity of SME-1†

**DOI:** 10.1039/d2ra02849b

**Published:** 2022-08-15

**Authors:** Vidhu Agarwal, Akhilesh Tiwari, Pritish Varadwaj

**Affiliations:** Indian Institute of Information Technology Devghat, Jhalwa, Prayagraj-211015 Allahabad U P India pritish@iiita.ac.in +919236666060

## Abstract

SME-1 is a carbapenemase, produced by *Serratia marcescens* organism and causes nosocomial infections such as in bloodstream, wounds, urinary tract, or respiratory tract infections. Treatment of such infections becomes very complex due its resistance towards penicillins, cephalosporins, monobactams, and carbapenems. Resistance to such antibiotics is of great medical concern. The misuse and overuse of these antibiotics result in the clinical mutation and production of novel β-lactamase enzymes such as SME-1, which show resistance to carbapenems. Class A contains most of the clinically significant extended spectrum of β-lactamase enzymes and carbapenemases. In this study, class A β-lactamase SME-1 sequence, structure, and binding were compared with naturally mutated class A β-lactamase enzymes and a wild-type TEM-1. This study was performed for revealing mutations, which could be responsible for the carbapenemase activity of SME-1. The dynamic characteristics of SME-1 enzymes manifest a different degree of conservation and variability, which confers them to possess carbapenemase activities. Met69Cys, Glu104Tyr, Tyr105His, Ala237Ser, and Gly238Cys mutations occur in SME-1 as compared to wild-type TEM-1. These mutated residues are present close to active site residues such as Ser70, Lys73, Ser130, Asn132, Glu166, and Asn170, which participate in the hydrolytic reaction of β-lactam antibiotics. Furthermore, these mutated residues demonstrate altered interactions with the β-lactam antibiotics (results in altered binding) and within themselves (results in active site structure alterations), which results in expanding the spectrum of activity of these enzymes. This study provides important insights into the structure and activity relationship of SME-1 enzymes. This is evident from the Ω-loop structure modification, which forms the wall of the active site and repositioning of residues involved in hydrolytic reactions, when present in the complex with meropenem in a stable state of MD simulation at 50 ns. Hence, Met69Cys, Glu104Tyr, Tyr105His, Ala237Ser, and Gly238Cys mutations could result in an altered active site structure, binding, and activity of SME-1 with meropenem and thus become resistantant against meropenem, which is a carbapenem.

## Abstract 2

Sequencing, molecular docking and dynamics, analysis of naturally mutated class A β-lactamase SME-1 was done to compared with 13 naturally mutated class A β-lactamase including SHV-1 and wild class A β-lactamase TEM-1.

## Graphical abstract

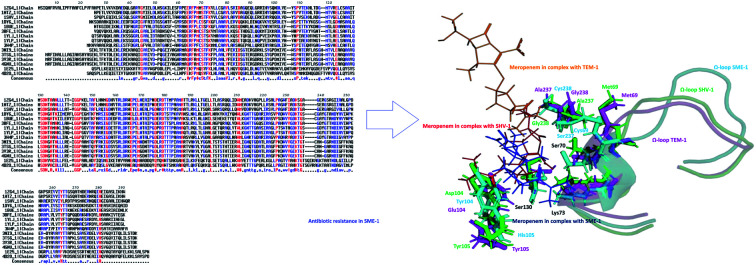

## Introduction

Carbapenems are broad-spectrum antibiotics, with enhanced stability against hydrolysis. The loss of the outer membrane permeability and the development of β-lactam hydrolyzing enzymes have been identified as one of the key mechanisms of carbapenem resistance. This results in one of the most resilient β-lactamase enzymes termed carbapenemase.^1^*Serratia marcescens* enzyme (SME) was identified as a class A carbapenemase, which was derived from two clinical strains of *Serratia marcescens* (in 1982) from England, Europe, North America, and South America. SME carbapenemase being atypical in nature, manifest a distinctive phenotypic profile, exhibiting tolerance to all β-lactams except extended-spectrum cephalosporins (ceftazidime and cefepime), including carbapenems and aztreonam.^2^*Serratia marcescens* causes community-acquired and nosocomial infections such as the circulation, wounds, urinary system, and respiratory tract. With the emergence of resistance, it has become extremely challenging to curb this pathogenic organism.^3,4^ The basic architecture and folds of the SME-1 structure are considerably comparable to those of class A beta-lactamase enzymes. Almost all the key active site residues positioned in SME-1 are conserved among class A β-lactamases except at 104, 105, and 237 loci, which are occupied by tyrosine, histidine, and serine, respectively.^5^ Cysteine at loci 238 forms a disulfide bridge with another cysteine positioned at 69 loci. The critical function of this disulfide bridge was substantiated by the site-directed substitution of Cys69 to Ala that produced an incapable variant that fails to impart resistance to imipenem and all other assayed β-lactams. The noteworthy structural characteristic of SME-1 was the positioning of the active site serine (70) residue and glutamate (166) by a minuscule distance of up to 1.4 nm compared to class A β-lactamases. As a result, the three-dimensional active site topology of SME-1 fails to accommodate the vital catalytic H_2_O molecules located between Ser70 characterized in class A beta-lactamases. This entails a significant conformational alteration in SME-1, which may be required for the appropriate positioning of the hydrolytic water molecule and directly implicated in the hydrolysis of the acyl-enzyme intermediate.^6^

The SME-1 carbapenemase Ser237Ala mutant displayed penicillin and aztreonam hydrolysis almost similar to wild-type class A β-lactamase enzyme but showed reduced susceptibility against cefoxitin, cephaloridine, and cephalothin. A sharp decline in the catalytic efficiency was observed against the imipenem, signifying the pertinent role of a serine residue in SME-1 carbapenemase activity.^6^ The significance of the disulfide linkage across Cys69 and Cys238 in the carbapenem hydrolysis, as well as other β-lactams, was also demonstrated. The loss of catalytic activity by the SME-1 carbapenemase Cys69Ala mutant against imipenem, cefoxitin, kanamycin, ticarcillin, amoxicillin, and aztreonam has been observed.^5^ PCR-based mutations have been used to generate different alternative libraries for both the Cys69 and Cys238 positions. Those enzymes from either of these libraries having Cys69 and Cys238 showed efficiency and competence in conferring resistance to β-lactams, indicating how these cysteines and the associated disulfide linkage are unfavorable to the hydrolysis of all β-lactam degraded by SME-1.^7^ Furthermore, SME-1 residues at 104, 105, 132, 167, 237, and 241 were subjected to randomised site-directed mutations, and proficient mutants were identified based on their capacity to hydrolyze imipenem, ampicillin, and cefotaxime. However, no specific site appeared essential for carbapenem hydrolysis, numerous locations appeared to be significant for β-lactam antibiotic hydrolysis, indicating that the carbapenemase activity of SME-1 is the consequence of a highly dispersed series of interactions that gradually modulates the conformation and architecture of the active site compartment.^8^

Functional insights into drug resistance can be found using MD simulation methods. Further, an atomic level of understanding can be gained using post-simulation methods such as PCA. This method enables the dimensionality reduction of different parameters of MD simulation in order to have an understanding of its concerted motion.^9^ PCA analysis can help in knowing the effect of mutation on the overall MD trajectory; hence, it is also known as essential dynamics.^10^ RIN and residual decomposition analysis help in knowing the effect of mutation on the protein residue interaction network and free energy change at the residue level, respectively.

This study focuses on determining the mutations responsible for the carbapenemase activity of SME-1 class A β-lactamase enzymes. This could be known from the comparison of sequence, structure, and interactions of SME-1 with naturally mutated and wild-type class A β-lactamase enzymes, in complex with β-lactam antibiotics. Further, molecular docking, MMGBSA, RMSD, and PCA analysis help in getting insights into the stability of SME-1 carbapenemase, as compared to naturally mutated and wild-type TEM-1 class A β-lactamase enzymes in complex with β-lactam antibiotics.

## Results

SME-1 has evolved clinically to extend its spectrum against the most effective and last resort of β-lactam antibiotics like carbapenems. Usually, the structures of these enzymes are conserved, including their active site residues such as Ser70, Lys73, Ser130, Asn132, Glu166, and Asn170 (for class A β-lactamase enzymes), which participate in the hydrolytic reaction with β-lactam antibiotics. The mutation of some important residues could alter the structure and activity of such β-lactamase enzymes.^11^ This could be due to alterations in the interaction pattern of the β-lactamase enzymes within themselves (active site structure alterations) and with β-lactam antibiotics (binding alteration), which could result in structural and functional modifications. These structural modifications caused due to mutations result in alterations in binding with the ligand and hence could be responsible for the carbapenemase activity of SME-1. Kinetic, molecular docking, molecular dynamic simulation, PCA, and MMGBSA analysis revealed altered interactions and stability of the SME-1 in complex with amoxicillin, ceftazidime, ceftolozane, and meropenem as compared to wild-type TEM-1 and naturally mutated class A β-lactamase enzymes.

### Kinetics analysis

Naturally mutated SME-1 class A β-lactamase is a carbapenemase.^12^ The catalytic efficiency of wild TEM-1 class A β-lactamase is 0.002 μM^−1^ s^−1^ as compared to the catalytic efficiency of clinically mutated SME-1 that is 0.44 μM^−1^ s^−1^ in complex with imipenem. Similarly, Table S1† shows the difference in catalytic efficiency of clinically mutated class A β-lactamase enzymes (SME-1 and SHV-1) and wild TEM-1 enzyme with respect to different β-lactam antibiotics. Further, from Table S1,† it is evident that the catalytic efficiency (*K*_cat_/*k*_m_) of SME-1 is higher, as compared to that of GES-1, GES-11, and GES-5, TEM-1 for cephalothin, cefoxitin and imipenem (carbapenem).

### Sequence analysis

**Fig. 1:**
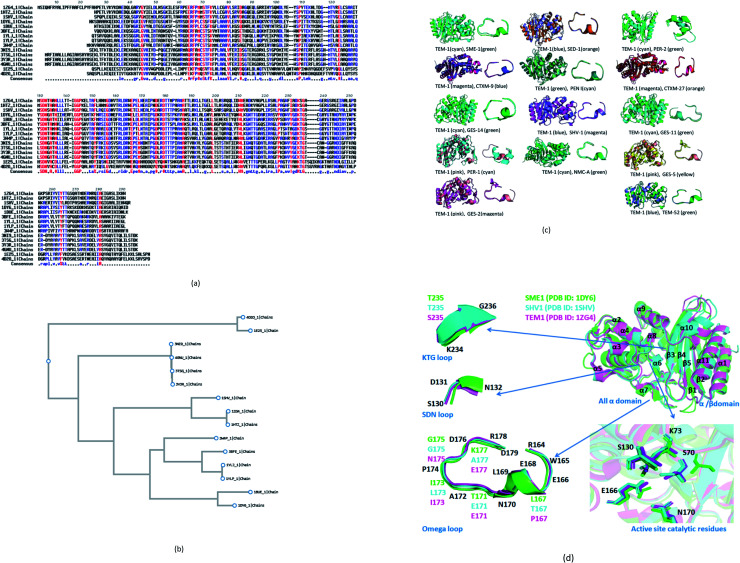
(a) Multiple sequence alignments of SME-1 (PDB ID: 1DY6) along with 13 naturally mutated class A β-lactamase enzymes (TEM-52 (PDB ID: 1HTZ), SHV-1 (PDB ID: 1SHV), SME-1 (PDB ID: 1DY6), NMC-A (PDB ID: 1BUE), SED-1 (PDB ID: 3BFE), CTXM-9 (PDB ID: 1YLJ), CTXM-27 (PDB ID: 1YLP), PENI (PDB ID: 3W4P), GES-2 (PDB ID: 3NI9), GES-11 (PDB ID: 3V3R), GES5 (PDB ID: 4GNU), PER-1 (PDB ID: 1E25) and PER-2 (PDB ID: 4D2O)) and TEM-1 (PDB ID: 1ZG4). (b) Shows the phylogenetic tree for the (PDB ID: 1DY6) along with 13 naturally mutated class A β-lactamase enzymes and TEM-1 (PDB ID: 1ZG4). (c) Shows overall and Ω-loop structure comparison of 14 naturally mutated class A β-lactamase enzymes (including SME-1) with wild type class A β-lactamase enzyme TEM-1. (d) Shows the comparison of SME-1 (carbapenemase), SHV-1 (penicillinase) and TEM-1 (wild type) class A β-lactamase enzyme.

14 naturally mutated and wild-type class A β-lactamase enzyme sequences were compared. Multiple sequence alignment results are shown in Fig. 1a. Furthermore, Table 1 combines the residues that could be indirectly involved in the alteration of β-lactam activity and the mutations present in 14 naturally mutated and wild-type class A β-lactamase enzyme sequences. Fig. 1b shows that TEM-1 (PDB ID: 1ZG4) and SHV-1 (PDB ID: 1SHV) are evolutionary very close. Further, SME-1 (PDB ID: 1DY6) is evolutionary very far from TEM-1 and SHV-1. As SHV-1 is a penicillinase (Table 1), it has a similar binding affinity with the TEM-1 enzyme and has a similar Ω-loop structure (Fig. 1c) and a lesser number of mutations on residues that could be indirectly involved in the alteration of the β-lactam activity, as compared to TEM-1 (Table 1). Therefore, for further analysis and comparison, the SHV-1 enzyme is selected as a naturally mutated class A β-lactamase enzyme, which can be compared with naturally mutated class A β-lactamase enzyme SME-1 with carabapenemase activity.

**Table 1:** Shows organism name, activity, PDB ID and mutations occurring in SME1 along with 13 clinically mutated and wild type class A β-lactamase enzymes and the colour highlights depicts Ω-loop structure modification: more difference (green), less difference (blue) and without difference (black), as compared to wild type TEM-1. Further, clinically mutated class A β-lactamase enzymes (SME-1 and SHV-1) and wild type class A β-lactamase enzyme (TEM-1) have been highlighted with bold

Organism name	Activity	PDB ID	Protein name	69	104	105	132	164	165	170	171	173	176	178	179	182	216	237	238	240	244	271	273	274
*Escherichia coli*	Wild type	1ZG4	TEM-1	Met	Glu	Tyr	Asn	Arg	Trp	Asn	Glu	Ile	Asp	Asn	Asp	Met	Val	Ala	Gly	Glu	Arg	Thr	Asp	Glu
*Klebsiellapneumoniae*	Cephalosporinase	1HTZ	TEM-52		Lys											Thr			Ser					
***Klebsiellapneumoniae***	**Penicillinase**	**1SHV**	**SHV-1**		**Asp**							**Leu**				**Thr**			**Gly**			**Ser**	**Ala**	
***Serratiamarscenes***	**Carbapenemase**	**1DY6**	**SME-1**	**Cys**	**Tyr**	**His**					**Thr**					**Thr**	**Thr**	**Ser**	**Cys**	**Ala**	**Ala**	**Asp**	**Lys**	**His**
*Enterobacter cloacae*	Carbapenemase	1BUE	NMC-A	Cys	Phe	His					Thr					Thr	Thr	Ser	Cys	Ala	Ala	Glu	Lys	His
*Cirobactersedlaki*	Cephlosporinase	3BFE	SED-1	Cys	Asn	Trp					Thr					Ser	Thr		Gly	Asp	Thr	Asp	Lys	Trp
*Escherichia coli*	Cephalosporinase	1YLJ	CTXM-9	Cys	Asn				Thr		Thr					Thr	Thr	Ser	Gly	Asp	Thr	Asn	Glu	Ser
*Escherichia coli*	Cephalosporinase	1YLP	CTXM-27	Cys	Asn				Thr		Thr					Thr	Thr	Ser	Gly	Gly	Thr	Asn	Glu	Ser
*Burkholderiapseudomallei*	Carbapenemase	3W4P	PENI	Cys	Arg				Lys		Thr	Leu				Thr	Thr	Thr	Gly	Asp	Ala	Asn	Gln	Ala
*Pseudomonas aeruginosa*	Carbapenemase	3NI9	GES-2	Cys		Trp			Lys		Asp	Thr				Thr	Thr	Thr	Cys	Asn		Ser	Val	
*Acinetobacterbaumannii*	Carbapenemase	3TSG	GES-14	Cys		Trp			Lys	Ser	Asp	Thr				Thr	Thr	Ser	Cys	Asn		Ser	Val	
*Acinetobacterbaumannii*	Carbapenemase	3V3R	GES-11	Cys		Trp			Lys	Gly	Asp	Thr				Thr	Thr	Ser	Cys	Asn		Ser	Val	
*Pseudomonas aeruginosa*	Carbapenemase	4GNU	GES-5	Cys		Trp			Lys	Ser	Asp	Thr				Thr	Thr	Thr	Cys	Asn		Ser	Val	
*Pseudomonas aeruginosa*	Cephalosporinase	1E25	PER-1	Gln	Thr	Trp		Ala	Asn	His	Ala	Asp	Gln	Gln	Asn	Ser	Thr	Thr	Ser	Lys	Thr	Ser	Arg	Thr
*Citrobacterfreundii*	Cephalosporinase	4D2O	PER-2	Gln	Thr	Trp		Ala	Asn	His	Ala	Asp	Gln	Gln	Asn	Ser	Thr	Thr	Ser	Lys	Thr	Ser	Arg	Thr

It can be observed from Table 1 that Glu104Tyr has a unique mutation in SME-1, as compared to other naturally mutated class A β-lactamase enzymes with respect to wild-type class A β-lactamase enzyme TEM-1. Tyr105His has a unique mutation, except for NMC-A compared to other clinically mutated class A β-lactamase enzymes with respect to wild-type class A β-lactamase enzyme TEM-1.

### Structure analysis

SME-1 is a carbapenemase. 14 naturally mutated and wild-type class A β-lactamase enzyme structures were aligned and visualized in PyMOL, as shown in Fig. 1c. Further, root mean square deviation (RMSD) calculations were also performed in PyMOL. It was performed on all 14 enzymes as compared to wild-type TEM-1 enzyme.

Class A β-lactamase enzymes contain two domains, between which the active site of the enzyme is present. The first domain contains eight α helix (α2–α9), whereas the second domain contains five β-strands (β1–β5) and three α helix (α1, α10, α11). Ser70, Lys73, Ser130, Asn132, Glu166, and Asn170 are conserved in all the naturally mutated class A β-lactamase enzymes and the wild-type class A β-lactamase enzyme. These residues participate in the hydrolytic reaction with β-lactam antibiotics including Ser70, which acts as a nucleophile and attacks the β-lactam ring for its hydrolysis.^13^ Ser70 was observed to be displaced in SME-1 by an RMSD value of 0.071, as compared to TEM-1. Ser70 residue in SME-1 is much more structurally recolated as compared to the wild TEM-1 and other clinically mutated class A β-lactamase enzymes. This is caused due to alterations in the active site structure of SME-1, due to clinical mutations as compared to wild TEM-1. It can be observed from Table 2 that Ser70 (0.071) and Ω-loop (0.366) deviation in SME-1 is greater with respect to wild class A β-lactamase enzyme TEM-1, as compared to other clinically mutated class A β-lactamase enzymes like NMC-A, PENI, GES-2, GES-5, GES-11 and GES-14. Further, Fig. 1d shows the structural relocation of Ser70 in SME-1, as compared to SHV-1 and TEM-1.

**Table 2:** The RMSD values for catalytically important residues, which directly participate in the catalytic hydrolysis of the β-lactam antibiotics and loops present in the active site of clinically mutated class A β-lactamase enzymes, as compared to the TEM-1 wild-type class A β-lactamase enzyme

S. No	Class A β-lactamase	Ω-loop	SDN-loop	Ser70	Lys73	Ser130	Glu166
1	SME1	0.366	0.154	0.071	0.164	0.156	0.051
2	PER2	2.010	0.164	0.045	0.636	0.193	0.058
3	TEM52	0.222	0.094	0.110	0.071	0.049	0.094
4	PENI	0.272	0.126	0.011	0.061	0.264	0.021
5	GES14	0.273	0.195	0.061	0.633	0.217	0.019
6	CTXM9	0.278	0.169	0.094	0.056	0.262	0.024
7	CTXM27	0.241	0.134	0.091	0.119	0.217	0.021
8	GES5	0.274	0.185	0.044	0.117	0.219	0.021
9	PER1	2.029	0.194	0.050	0.089	0.236	0.125
10	SED1	0.372	0.170	0.286	0.173	0.517	0.063
11	GES11	0.333	0.225	0.047	0.632	0.275	0.0402
12	NMCA	0.316	0.178	0.067	0.148	0.027	0.022
13	GES2	0.287	0.236	0.034	0.137	0.203	0.026
14	SHV1	0.212	0.081	0.031	0.041	0.025	0.014

Ω-loop and SDN loop forms the wall of the active site and is the most flexible element. Any structural modification in the active site can be easily reflected by the Ω-loop structure modification. 6 Carbapenemases NMC-A (0.316), PENI (0.272), GES-2 (0.287), GES-5 (0.274), GES-11 (0.333) and GES-14 (0.273) have a lower RMSD deviation in the Ω-loop structure, as compared to the Ω-loop structure of naturally mutated SME-1 (0.366) with respect to wild type TEM-1. Among the carbapenemases, SME-1 has the greatest deviation in the Ω-loop and SDN loop structure, as compared to wild-type TEM-1. This can be observed from the data in Fig. 1c and d, and Table 2.

### Molecular docking and MMGBSA

The MMGBSA dG bind value gives insights into the binding affinity between the protein and ligand in the docked complex. It is evident from Table 3 that SME-1 has minimum binding energy (MMGBSA dG bind: −41.361 kcal mol^−1^) and hence maximum binding affinity for meropenem (carbapenem), as compared to the other 13 naturally mutated class A β-lactamase enzymes and wild-type class A β-lactamase enzymes. Alterations in the binding and affinity of 14 naturally mutated class A β-lactamase enzymes including SME-1 carbapenemase and TEM-1 wild-type class A β-lactamase in the complex with different β-lactam antibiotics evident from the data in Table 3. SME-1 is a carbapenemase and hence has a greater binding affinity (−41.361 kcal mol^−1^), as compared to TEM-1 (−32.69 kcal mol^−1^) in the complex with meropenem. Further, SME-1 binding affinity is greater depicted by MMGBSA dG bind values (−34.84, −37.85, and −62.68 kcal mol^−1^), as compared to TEM-1 (−34.53, −33.78, and −15.72 kcal mol^−1^) in complex with amoxicillin, ceftazidime, and ceftolozane as well. This shows that SME-1 is truly a carbapenemase, as it has a greater binding affinity for amoxicillin, ceftazidime, ceftolozane, and meropenem, as compared with TEM-1. Other carbapenemases do not follow a similar binding pattern with amoxicillin, ceftazidime, ceftolozane, and meropenem, except GES-5. GES-5 is a carbapenemase (Table 1) and hence it has a greater binding affinity with amoxicillin (−42.59 kcal mol^−1^), ceftazidime (−56.46 kcal mol^−1^), and ceftolozane (−37.45 kcal mol^−1^), as compared to TEM-1 (Table 3).

**Table 3:** Class A β-lactamase enzymes with their docking and MMGBSA energy scores, as compared with wild-type TEM1 in complex with amoxicillin, ceftazidime, ceftolozane, and meropenem. This depicts the difference in the binding affinity of different clinically mutated class β-lactamase enzymes, derived from different organisms that show the difference in β-lactam antibiotic binding

Organism	Protein (class A β-lactamase enzyme)-ligand (β-lactam antibiotics)	PDB ID of class A β-lactamase enzyme	Docking score	Glide gscore	Glide energy	Glide emodel	MMGBSA dG Bind	MMGBSA dG Bind Coulomb	MMGBSA dG Bind Covalent	MMGBSA dG Bind Hbond	MMGBSA dG Bind Solv GB	MMGBSA dG Bind vdW
***Serratiamarscenes***	**SME1**	**1DY6**										
	**Amoxicilline**		**−4.942**	**−5.134**	**−41.488**	**−49.541**	**−34.84**	**−26.72**	**4.7**	**−0.93**	**38.14**	**−33.44**
	**Ceftazidime**		**−5.291**	**−5.291**	**−49.394**	**−62.62**	**−37.85**	**−22.74**	**5.61**	**−2.06**	**48.93**	**−47.12**
	**Ceftolozane**		**−6.341**	**−6.342**	**−52.602**	**−81.153**	**−62.68**	**−10.32**	**4.29**	**−8.5**	**22**	**−43.51**
	**Meropenem**		**−4.857**	**−4.886**	**−40.394**	**−54.498**	**−41.361**	**−6.32**	**1.579**	**−1.577**	**13.069**	**−37.487**
*Cirobactersedlaki*	SED1	3BFE										
	Amoxicilline		−7.415	−8.181	−37.841	−49.037	−36.32	−45.35	2.04	−4.73	52.34	−26.41
	Ceftazidime		−4.492	−4.492	−48.263	−65.276	−57.02	−8.64	−0.65	−2.95	21.91	−38.39
	Ceftolozane		−6.458	−6.46	−59.385	−85.323	−46.9	−21.79	6.3	−4.96	36.73	−39.93
	Meropenem		−4.136	−4.165	−33.83	−44.618	−23.305	−0.325	2.211	−1.981	26.494	−36.604
*Burkholderiapseudomallei*	PENI	3W4P										
	Amoxicilline		−3.627	−3.82	−32.541	−46.383	−22.78	−23.36	7.14	−4.21	41.94	−26.4
	Ceftazidime		−3.953	−3.953	−55.889	−73.136	−27.91	−11.72	10.12	−3.41	51.55	−46.25
	Ceftolozane		−7.981	−7.982	−67.92	−93.033	−54.3	34.84	6.73	−4.92	−20.19	−47.07
	Meropenem		−6.124	−6.153	−36.822	−44.807	−39.215	−24.305	3.447	−2.518	27.643	−31.139
*Acinetobacterbaumannii*	GES14	3TSG										
	Amoxicilline		−4.013	−4.205	−38.355	−48.642	−30.32	114.82	8.22	−0.89	−103.2	−27.71
	Ceftazidime		−4.517	−4.517	−40.457	−48.753	−33.53	144.09	8.89	−2.39	−115.08	−43.83
	Ceftolozane		−6.895	−6.897	−58.641	−81.308	−69.42	7.17	6.91	−4.84	5.32	−53.53
	Meropenem		−3.958	−3.987	−32.504	−42.522	−13.615	55.736	2.849	−0.815	−31.539	−30.15
*Acinetobacterbaumannii*	GES11	3V3R										
	Amoxicilline		−5.066	−5.831	−38.753	−45.828	−38.23	−19.54	5.41	−1.54	23.16	−25.73
	Ceftazidime		−5.398	−5.398	−37.045	−47.583	−37.59	37.01	0.63	−5.56	−20.3	−32.62
	Ceftolozane		−8.738	−8.739	−63.906	−96.889	−82.99	6.68	−0.45	−10.31	−9.36	−47.13
	Meropenem		−4.304	−4.333	−38.314	−46.366	−21.596	23.909	8.066	−0.526	−5.268	−35.522
*Citrobacterfreundii*	PER2	4D2O										
	Amoxicilline		−4.99	−5.183	−47.685	−62.932	−47.01	0.37	3.26	−1.29	14.18	−40.56
	Ceftazidime		−7.391	−7.391	−60.736	−79.905	−71.68	−16.27	9.84	−2.91	21.72	−51.24
	Ceftolozane		−7.424	−7.425	−67.906	−94.476	−77.89	1.09	10.24	−9.35	7.75	−53.22
	Meropenem		−4.439	−4.468	−40.214	−51.792	−33.47	10.138	4.783	−1.129	8.124	−43.564
*Escherichia coli*	CTXM9	1YLJ										
	Amoxicilline		−5.265	−6.03	−42.948	−53.93	−34.04	−62.42	8.68	−5.09	63.49	−24.3
	Ceftazidime		−4.093	−4.093	−36.929	−44.897	−33.99	84.03	0.25	−1.79	−56.75	−32.52
	Ceftolozane		−9.379	−9.38	−59.672	−75.677	−61.27	−24.97	−6.38	−8.71	20.3	−32.37
	Meropenem		−5.518	−5.547	−37.452	−46.731	−19.007	−8.192	6.083	−2.741	15.283	−20.677
*Escherichia coli*	CTXM27	1YLP										
	Amoxicilline		−4.117	−4.882	−37.016	−45.379	−41.21	−45.76	1.56	−1.18	45.43	−27.09
	Ceftazidime		−5.364	−5.364	−46.69	−56.532	−40.85	13.55	5.63	−1.44	−1.09	−40.46
	Ceftolozane		−7.156	−7.158	−57.214	−69.598	−71.1	−54.77	4.4	−6.63	48.98	−40.75
	Meropenem		−5.029	−5.058	−33.846	−42.849	−27.498	−11.109	1.58	−2.402	22.535	−30.742
***Escherichia coli***	**TEM1**	**1ZG4**										
	**Amoxicilline**		**−6.437**	**−7.203**	**−50.66**	**−54.842**	**−34.535606**	**−20.088852**	**3.132864**	**−4.374707**	**34.175854**	**38.996207**
	**Ceftazidime**		**−3.541**	**−3.541**	**−46.618**	**−50.295**	**−33.784927**	**48.923797**	**7.830532**	**−1.263179**	**−24.075503**	**−48.803743**
	**Ceftolozane**		**−5.845**	**−5.847**	**−53.88**	**−72.924**	**−15.725121**	**6.46532**	**7.521848**	**−5.716508**	**33.881889**	**−49.645526**
	**Meropenem**		**−3.799**	**−3.828**	**−41.673**	**−54.431**	**−32.69**	**1.2**	**3.647**	**−2.021**	**17.335**	**-41.025**
*Enterobacter cloacae*	NMCA	1BUE										
	Amoxicilline		−4.582	−4.775	−38.645	−44.203	−33.75	26.13	0.89	−1.17	−11.33	−33.4
	Ceftazidime		−3.718	−3.718	−44.239	−55.188	−22.63	43.05	−0.89	−2.32	−4.5	−40.23
	Ceftolozane		−4.668	−4.67	−56.137	−75.439	−51.59	33	3.92	−3.04	−6.49	−54.72
	Meropenem		−4.167	−4.196	−33.267	−41.508	−29.996	4.995	6.768	−1.698	4.119	−34.295
*Klebsiellapneumoniae*	TEM52	1HTZ										
	Amoxicilline		−6.332	−7.097	−40.46	−47.087	−33.308 376	−11.446 065	−2.479 853	−3.685 972	30.414 074	−39.09642
	Ceftazidime		−5.863	−5.863	−48.459	−58.224	−35.548 678	45.387 819	1.8867	−4.815 511	−30.422 939	−35.844 801
	Ceftolozane		−7.728	−7.729	−63.368	−89.607	−43.727 577	−8.678 022	12.599 606	−7.620 953	15.554 243	−44.924 203
	Meropenem		−4.58	−4.609	−36.306	−48.323	−39.535	1.909	4.728	−1.71	7.148	−40.46
**Klebsiellapneumoniae**	**SHV1**	**1SHV**										
	**Amoxicilline**		**−3.501**	**−3.694**	**−36.025**	**−43.964**	**−30.387442**	**−41.393498**	**2.315058**	**−3.007014**	**51.627833**	**−30.977501**
	**Ceftazidime**		**−4.014**	**−4.014**	**−43.961**	**−49.525**	**−39.270824**	**−17.56272**	**8.542979**	**−2.90362**	**31.825936**	**−46.806289**
	**Ceftolozane**		**−4.558**	**−4.559**	**−65.063**	**−81.931**	**−28.856741**	**−24.442419**	**10.976806**	**−6.497273**	**56.030517**	**−53.672098**
	**Meropenem**		**−3.319**	**−3.348**	**−30.939**	**−38.68**	**−30.816**	**−25.064**	**0.772**	**−2.009**	**39.061**	**−33.654**
*Pseudomonas aeruginosa*	PER1	1E25										
	Amoxicilline		−6.151	−6.916	−46.751	−65.182	−26.33	7.98	−4.26	−1.07	−1.48	−13.23
	Ceftazidime		−4.978	−4.978	−51.766	−67.484	−27.03	−17.43	0.57	−1.04	16.12	−8.44
	Ceftolozane		−8.214	−8.215	−58.015	−79.262	−18.68	9.03	−0.59	−0.01	−1.04	−12.67
	Meropenem		−5.586	−5.615	−40.571	−57.362	−41.03	−14.776	0.931	−1.6	26.921	−41.567
*Pseudomonas aeruginosa*	GES2	3NI9										
	Amoxicilline		−3.645	−4.41	−32.32	−39.426	−38.16	−6.57	2.43	−6.02	14.51	−26.66
	Piperacillin		−1.975	−1.977	−40.605	−43.059	−42.43	−5.57	13.47	−4.27	12.07	−39.81
	Ceftazidime		−2.916	−2.916	−40.856	−53.222	−30.55	−2.99	4.84	−4.77	15.53	−29.87
	Ceftolozane		−3.966	−3.968	−49.424	−56.246	−51.69	−8.77	10.3	−11.62	18.69	−39.09
	Meropenem		−3.051	−3.08	−25.021	−27.526	−22.62	−8.559	5.576	−2.225	10.384	−23.373
*Pseudomonas aeruginosa*	GES5	4GNU										
	Amoxicilline		−4.833	−5.598	−41.477	−52.856	−42.59	−21.55	1.89	−1.71	32.72	−31.6
	Ceftazidime		−3.862	−3.862	−38.618	−47.176	−56.46	−18.02	0.74	−7.64	28.07	−37.81
	Ceftolozane		−5.386	−5.387	−48.971	−73.875	−39.27	30.95	0.62	−5.32	−4.51	−39.02
	Meropenem		−3.352	−3.381	−37.002	−48.355	−37.45334	−6.317 118	2.545 355	−1.588 386	13.09869	−34.729 868

SED1 is a cephalosporinase (Table 1) and hence its binding affinity is greater as depicted by MMGBSA dG binding values (−36.32, −57.02, and −46.9 kcal mol^−1^), as compared to TEM-1 (−34.53, −33.78, and −15.72 kcal mol^−1^) in complex with amoxicillin, ceftazidime, and ceftolozane, respectively. However, in SED-1, the binding affinity is lower (−23.305), as compared to TEM-1 (−32.69 kcal mol^−1^) in the complex with meropenem. Similar is the case of CTXM-9, CTXM-27, and to some extent NMC-A and PER2, but not with PER1, which all are cephalosporinase class A β-lactamase enzymes (Table 3).

**Fig. 2:**
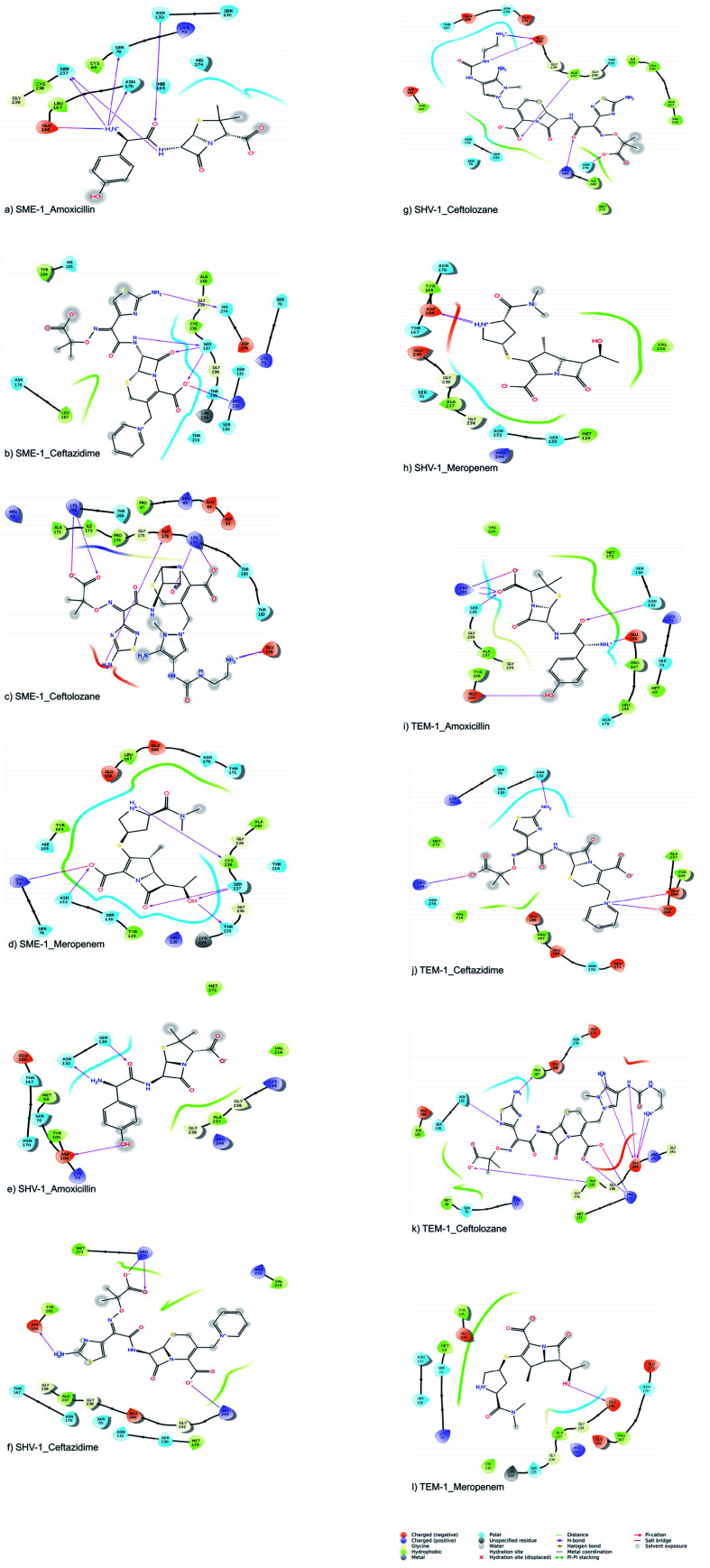
Shows the interaction diagram after docking between (a) SME-1_Amoxicillin (b) SME-1_Ceftazidime (c) SME-1_Ceftolozane (d) SME-1_Meropenem (e) SHV-1_Amoxicillin (f) SHV-1_Ceftazidime (g) SHV-1_Ceftolozane (h) SHV-1_Meropenem (i) TEM-1_Amoxicillin (j) TEM-1_Ceftazidime (k) TEM-1_Ceftolozane (l) TEM-1_Meropenem.

The molecular interactions between the active sites of class A β-lactamase enzymes (SME-1, SHV-1, and TEM-1) and β-lactam antibiotics (amoxicillin, ceftazidime, ceftolozane, and meropenem) were analyzed. In SME-1, Tyr104 does not form any hydrogen bond but is involved in a hydrophobic interaction with ceftazidime and meropenem (Fig. 2a and d). Asp104 of SHV-1 and Glu104 of TEM-1 form a hydrogen bond with amoxicillin, ceftazidime, and meropenem (Fig. 2e and f, Fig. 2h and i, Fig. 2j and l, respectively). This shows that Glu104Tyr mutation in SME-1 plays an important role in altering its binding with amoxicillin, ceftazidime, and meropenem. In SME-1, His105 is involved in polar interaction with amoxicillin, ceftazidime, and meropenem (Fig. 2a and b, and Fig. 2d), respectively. However, in TEM-1 and SHV-1, Tyr105 forms hydrophobic interactions with amoxicillin, ceftazidime, ceftolozane, and meropenem (Fig. 2e–l). This shows that the Tyr105His mutation in SME-1 alters the interaction with amoxicillin, ceftazidime, and meropenem. Glu171 forms an ionic bond with Arg164 and Arg178, which results in an inward turn in the Ω-loop.^13^ SME-1 contains Glu171Thr mutation (compared to TEM-1), resulting in the disruption of this ionic bond between Arg164, Arg178, and Glu171. The Glu171Thr mutation in SME-1 has a role in altering binding with meropenem (Fig. 2d and l). Thr216 forms a polar interaction in SME-1, whereas Val216 forms hydrophobic interaction in TEM-1, with ceftazidime and meropenem (Fig. 2b, d, j and l). Hence, Val216Thr forms altered interactions in the case of SME-1 with ceftazidime and meropenem, as compared to TEM-1. Two hydrogen bonds are formed between the Ser237 residue of SME-1 and amoxicillin, ceftazidime, and meropenem (Fig. 2a, b and d, respectively). In contrast, no hydrogen bond formation was observed between the Ala237 residue of TEM-1 and SHV-1 with amoxicillin, ceftazidime, and meropenem (Fig. 2e, f, h–j, and 2l). Hence, Ala237Ser mutation could be responsible for altered interactions between SME-1 and amoxicillin, ceftazidime, and meropenem, as compared to the interaction between wild type TEM-1 and amoxicillin, ceftazidime, and meropenem. One hydrogen bond is formed between the Cys238 residue of SME-1 and meropenem (Fig. 2d), whereas no hydrogen bond is formed between the Gly238 residues of TEM-1 and SHV-1 with meropenem (Fig. 2h and l). Hence, Gly237Cys mutation could be responsible for altered interactions of SME-1 with meropenem, as compared to the interaction between wild type TEM-1 and amoxicillin, ceftazidime, and meropenem. In SME-1, no hydrogen bond is formed between Ala240 and amoxicillin, ceftazidime, ceftolozane, and meropenem (Fig. 2a–d), but in TEM-1, Glu240 forms a salt bridge with ceftazidime (Fig. 2j), four hydrogen bond with ceftolozane (Fig. 2k) and one hydrogen bond with meropenem (Fig. 2l). Hence, Glu240Ala mutation alters interaction in SME-1 with ceftazidime, ceftolozane, and meropenem, as compared with TEM-1.

### Molecular dynamic simulation

#### Root mean square deviation (RMSD) analysis

**Fig. 3:**
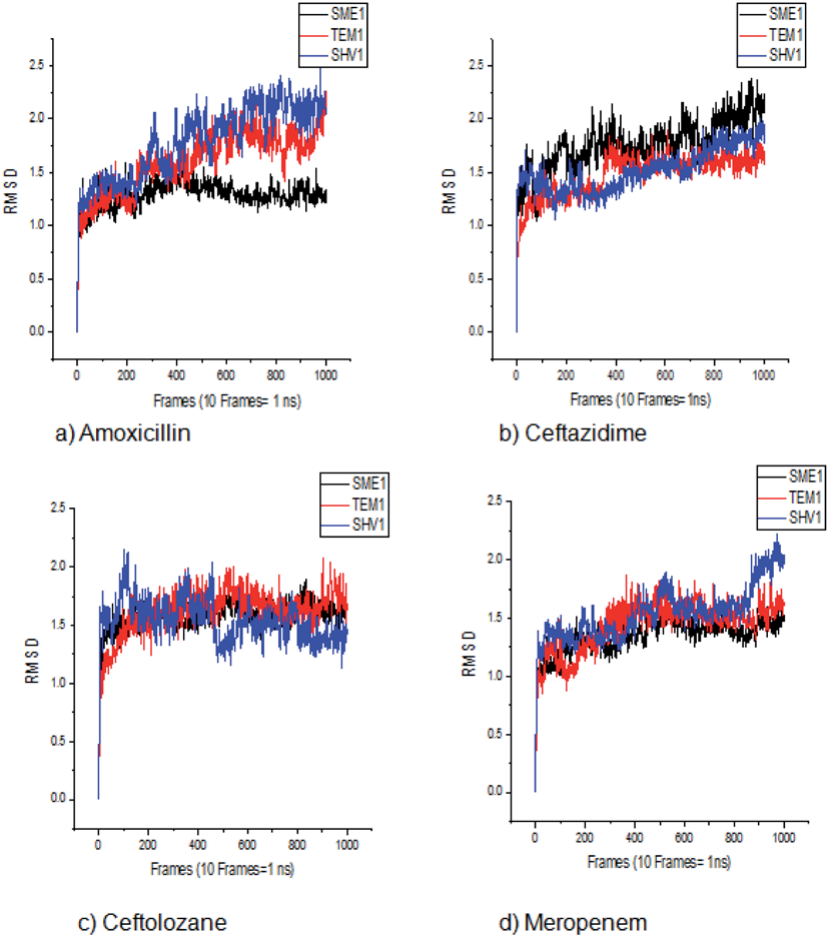
The RMSD plot of clinically mutated class A β-lactamase enzymes (SME-1 and SHV-1) and wild class A β-lactamase enzyme TEM-1 in complex with (a) amoxicillin (b) ceftazidime (c) ceftolozane (d) meropenem.

The RMSD calculations can give an insight into the structural conformation change, throughout the simulation time. The global RMSD of the backbone atom was below 2.5 Å for 100 ns, showing the overall stability and absence of significant conformational change in all the cases. The mean RMSD value of SME-1 (1.110) is less, compared with SHV-1 (1.532) and TEM-1 (1.283) with meropenem. The values are tabulated in Table S2.† Furthermore, Fig. 3 and the above values suggest that the naturally mutated class A β-lactamase enzyme SME-1 has a better binding with meropenem and amoxicillin, as compared to SHV-1 and TEM-1.

#### Root mean square fluctuation (RMSF) analysis

**Fig. 4:**
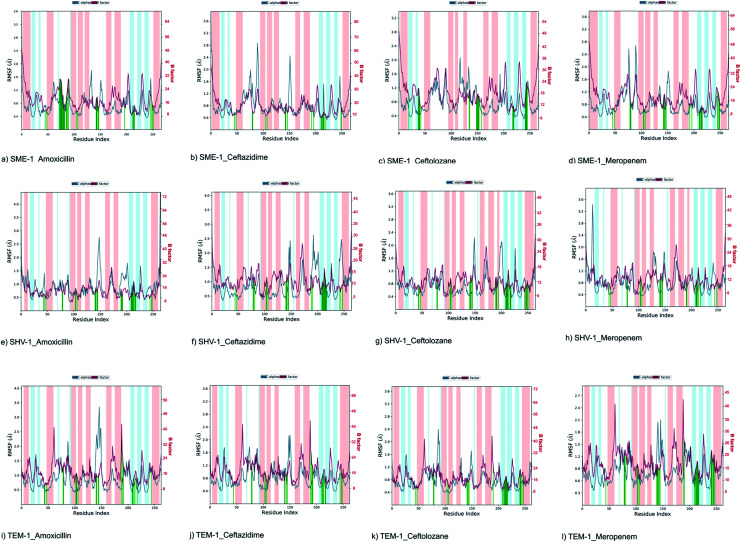
Shows the RMSF plot and B factor plot of (a) SME-1_Amoxicillin (b) SME-1_Ceftazidime (c) SME-1_Ceftolozane (d) SME-1_Meropenem (e) SHV-1_Amoxicillin (f) SHV-1_Ceftazidime (g) SHV-1_Ceftolozane (h) SHV-1_Meropenem (i) TEM-1_Amoxicillin (j) TEM-1_Ceftazidime (k) TEM-1_Ceftolozane (l) TEM-1_Meropenem complex. Alpha helix (represented by the orange background of the plot), and beta-sheet (represented by the light blue background of the plot) are the loop regions (represented by the white background of the plot). Green lines show the residues of protein that interact with the ligand.

The RMSF plot (Fig. 4) shows the fluctuation in atomic residues of the protein. The peaks of the plots depict the maximum fluctuation. Alpha helix (represented by an orange background of the plot) and beta-sheet (represented by a light blue background of the plot) are the more rigid parts of the protein, as compared to the loop (represented by the white background of the plot). Ω-loop plays an important role in the positioning of Glu166, which is involved in the hydrolytic reaction of the β-lactam ring present in the antibiotic substrate. Further, Ω-loop forms the wall of the active site, and hence its flexibility may indicate any structural and conformational modification in the active site. The Ω-loop (residue 164–179) flexibility was decreased in SME-1 in complex with β-lactam antibiotics (amoxicillin, ceftazidime, ceftolozane, and meropenem), as compared to that with TEM-1. This is evident from the data in Table 2, Table 4, and Fig. 4.

**Table 4:** RMSF values calculated for clinically mutated class A β-lactamase enzymes (SME-1 and SHV-1) and wild class A β-lactamase (TEM-1) in complex with meropenem

Residue	SME-1	SHV-1	TEM-1
Ser70	0.755	0.378	0.43
Lys73	0.457	0.348	0.39
Ser130	0.649	0.357	0.427
Asp131	0.544	0.334	0.362
Asn132	0.572	0.32	0.379
Arg164	0.75	0.398	0.493
Trp165	1.019	0.4	0.523
Glu166	0.859	0.406	0.517
Glu168	0.794	0.476	0.561
Leu169	0.686	0.388	0.476
Asn170	0.756	0.438	0.469
Ala172	0.859	0.69	0.57
Pro174	2.795	2.654	1.295
Asp176	2.085	1.581	0.924
Arg178	1.077	0.629	0.611
Asp179	0.642	0.432	0.461

### Interaction analysis

SME-1 and SHV-1 are naturally mutated class A β-lactamase and have different binding and activity towards amoxicillin, ceftazidime, ceftolozane, and meropenemas, per the molecular docking and molecular dynamic simulations results. This analysis can help us to gain insight into the altered active site residue interactions. According to the molecular docking and molecular dynamics simulation studies, SME-1 has a good binding affinity throughout the simulation of 100 ns with meropenem, as compared to TEM-1 and SHV-1 (Table 2 and Fig. 3).

**Fig. 5:**
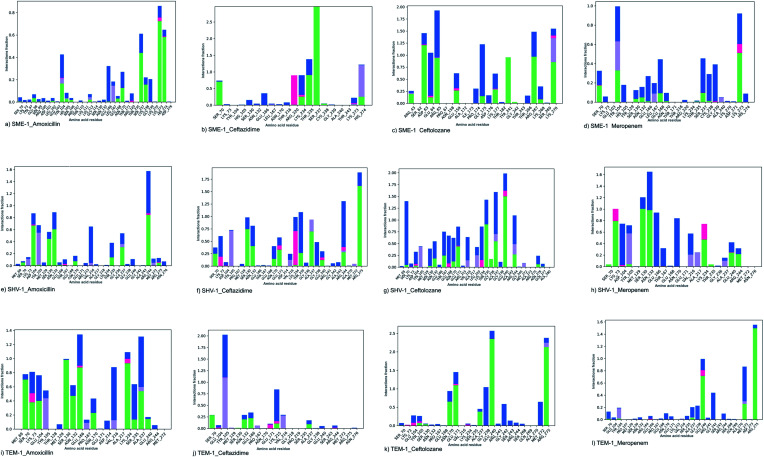
The interaction fraction plot of amino acids between (a) SME-1_Amoxicillin (b) SME-1_Ceftazidime (c) SME-1_Ceftolozane (d) SME-1_Meropenem (e) SHV-1_Amoxicillin (f) SHV-1_Ceftazidime (g) SHV-1_Ceftolozane (h) SHV-1_Meropenem (i) TEM-1_Amoxicillin (j) TEM-1_Ceftazidime (k) TEM-1_Ceftolozane (l) TEM-1_Meropenem.

It was observed that Tyr104 (in SME-1) has an increased interaction fraction with amoxicillin and meropenem (Fig. 5a and d), as compared to Asp104 (in SHV-1) interaction fraction and Glu104 (in TEM-1) interaction fraction with all the β-lactam antibiotics considered in the study, including meropenem (Fig. 5).

SME-1, Ser237, and Cys238 show significant interaction with meropenem, whereas Ala237 and Gly238 (in SHV-1 and TEM-1) do not interact much with meropenem (Fig. 5d, h, and l).

**Fig. 6:**
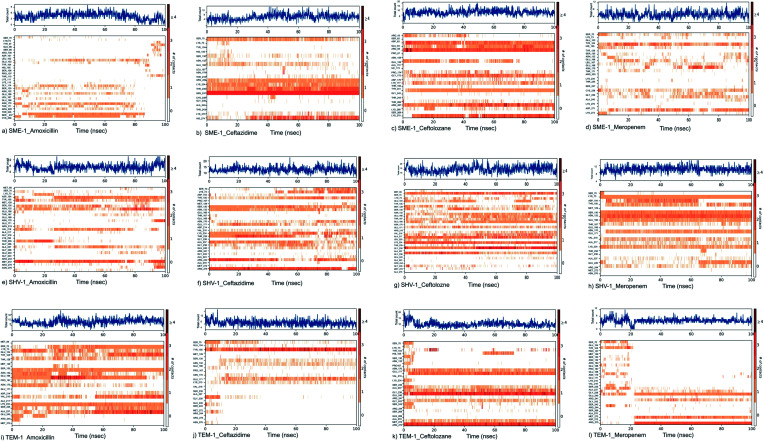
Shows the timeline representation of interaction and contact between (a) SME-1_Amoxicillin (b) SME-1_Ceftazidime (c) SME-1_Ceftolozane (d) SME-1_Meropenem (e) SHV-1_Amoxicillin (f) SHV-1_Ceftazidime (g) SHV-1_Ceftolozane (h) SHV-1_Meropenem (i) TEM-1_Amoxicillin (j) TEM-1_Ceftazidime (k) TEM-1_Ceftolozane (l) TEM-1_Meropenem.

Tyr104 in SME-1 forms a significant and constant interaction for 100 ns, which is absent in the case of SHV-1 and TEM-1 in complex with meropenem (Fig. 6d, h, and l). His105 shows slight interaction with SME-1 after 25 ns, with meropenem (Fig. 6d) but no interaction with TEM-1 after 25 ns, or meropenem (Fig. 6l).

### Principal component analysis

**Fig. 7:**
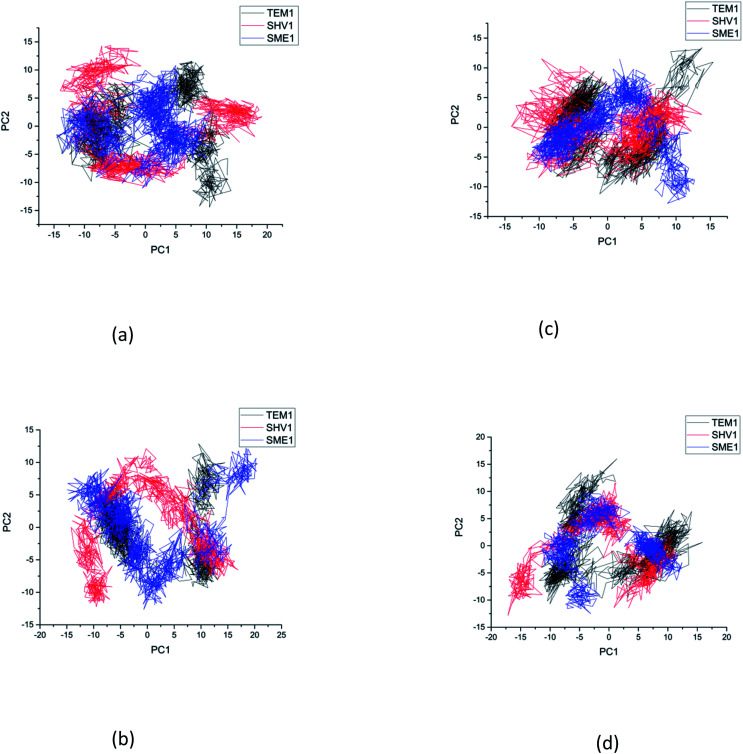
The principal component analysis of clinically mutated class A β-lactamase enzymes (SME-1 and SHV-1) and wild class A β-lactamase enzyme TEM-1 with (a) amoxicillin (b) ceftazidime (c) ceftolozane (d) meropenem.

Principal component analysis (PCA) was performed to understand the correlation between the secondary structure fragments of the complex along with the contribution of its residues. This could help in gaining insights into the protein complex dynamics by analyzing the coefficients of the principal components.^14^ Protein biological function is governed by protein conformations and their dynamics. A functional protein demonstrates flexibility and rigidity of its constituent residues to a different extent. Protein-ligand stability means that there is a tighter interaction between the active site residues of the protein and the ligand. Further, it could also mean that this complex becomes stable and its motion is restricted to some extent. Thus, the conformation of the biologically active proteins results in activity. Hence, for understanding the collective motion of the protein-ligand complex present in the conformational space for the molecular dynamics simulations, the dimension reduction method or the essential dynamic calculations is an appropriate analysis for projecting principal component 1 (PC2) and principal component 2 (PC2). This calculation was performed by diagonalizing the covariance matrix of the eigenvectors, for understanding the subspaces in which most of the protein dynamics occur.^15^ The PCA plot of SME-1, SHV-1, and TEM-1 in complex with amoxicillin, ceftazidime, ceftolozane, and meropenem shows that SME-1 is more stable, compared with SHV-1 and TEM-1 with meropenem and amoxicillin (Fig. 7). This observation leads to the conclusion that mutations in SME-1, as compared to TEM-1 and SHV-1, result in better binding with ligands such as amoxicillin and meropenem. RMSD plot also shows that SME-1 has a better binding with amoxicillin and meropenem, as compared to those in TEM-1 and SHV-1. This means that the mutations that occur uniquely in SME-1, as compared to in TEM-1 and SHV-1 could be responsible for altering the binding properties of SME-1 with amoxicillin and meropenem.

### Residue interaction analysis

**Fig. 8:**
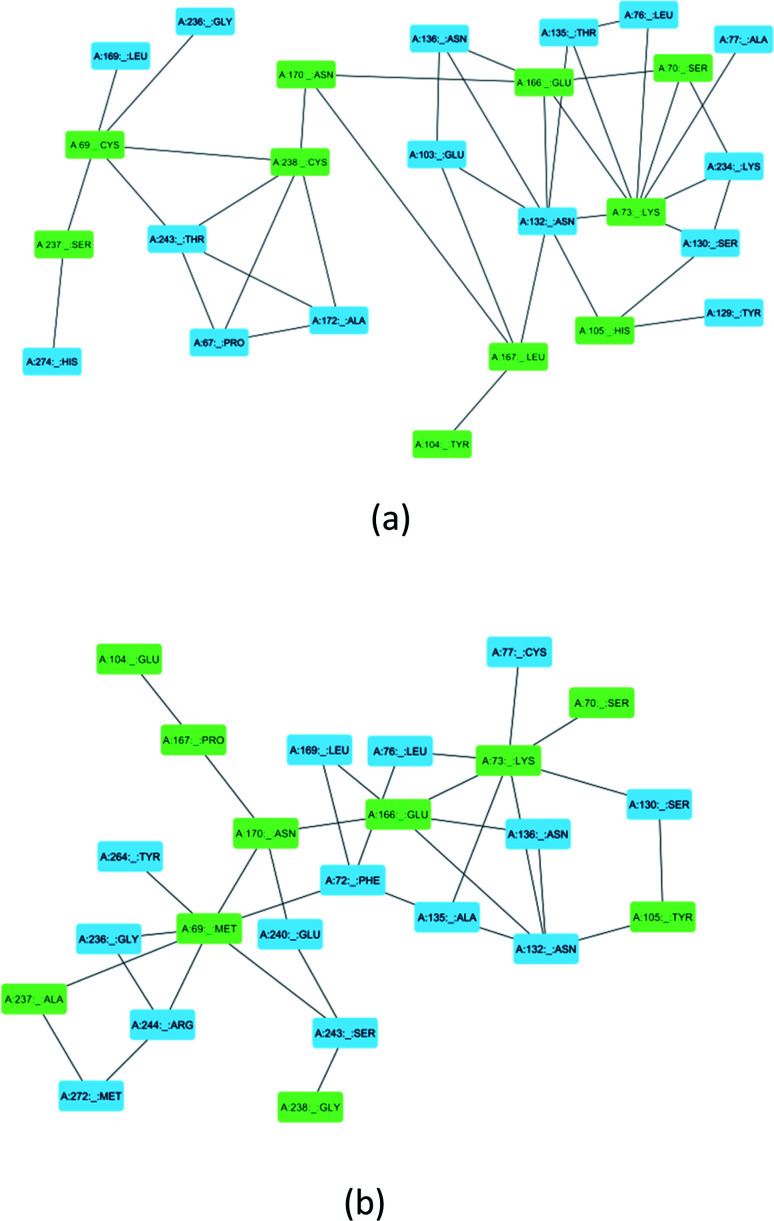
(a) Shows the residue interaction network of the SME-1 enzyme (b) Shows the residue interaction network of TEM-1 enzyme. The green colour shows mutated residues along with the residues of catalytic importance and the blue colour shows its neighbouring residues that directly interact with mutated residues.

Ser70, Lys73, Ser130, Asn132, Glu166, and Asn170 are residues that directly participate in the catalytic hydrolysis of β-lactam antibiotics. However, several other important residues are uniquely mutated in SME-1, which could play an important role in altering the structure of the enzyme and thus are responsible for carbapenemase activity. This could be due to the local structural modification, which results from the altered interactions and hydrogen bonding patterns with β-lactam antibiotics. Fig. 8 shows the modification in the interaction pattern in SME-1, as compared to that in TEM-1. Fig. 8a shows the interaction between mutated residues with their closest associated residues in SME-1, whereas Fig. 8b shows the same for the TEM-1 enzyme. It can be observed that residues 69, 104, 105, 237, and 238 are mutated in SME1, as compared to the wild-type TEM-1 enzyme. These mutated residues are closely connected with Ser70, Lys73, Ser130, Asn132, Glu166, and Asn170 residues, which participate in the hydrolytic reactions of β-lactam antibiotics.

### MMGBSA calculations of the MD trajectory

To explore the effect of clinical mutations in SME-1 compared to TEM-1 on binding with meropenem, MMGBSA of MD trajectory of class A β-lactamase enzymes (SME-1 and TEM-1) with β-lactam antibiotics were calculated. The average dGbind is lower (−75.04 087 792 kcal mol^−1^ and −59.0031 kcal mol^−1^) for SME-1, as compared with TEM-1 (−68.8181 kcal mol^−1^ and −56.7565 kcal mol^−1^) in the complex with amoxicillin and meropenem, respectively. This shows that SME-1 is more stable, as compared to SHV-1 and TEM-1 with meropenem and amoxicillin (Table 5).

**Table 5:** Shows the MMGBSA calculations of the SME-1 and TEM-1 MD trajectory in the complex with meropenem

Protein (Class A β-lactamase)	Ligand (β-lactam antibiotics)	MMGBSA dG Bind	MMGBSA dG Bind Coulomb	MMGBSA dG Bind Covalent	MMGBSA dG Bind Hbond	MMGBSA dG Bind Solv GB	MMGBSA dG Bind vdW
SME-1	Amoxicillin	−75.04 087 792	−16.22 674 604	5.636 274 192	−9.607 015 983	21.21 115 165	−48.61 465 048
	Ceftazidime	−83.5186	−75.4778	3.86303	−5.550 947 299	65.95 994 743	−49.43 714 579
	Ceftolozane	−70.3896	14.8433	1.83726	−7.30828	−1.57884	−50.367
	Meropenem	−59.0031	−35.0996	5.099636	−0.66614	50.0101	−40.6197
TEM-1	Amoxicillin	−68.8181	−80.0014	12.93065	−9.30847	68.84899	−39.8453
	Ceftazidime	−84.4038	−37.7148	0.816 403	−1.64518	40.76461	−50.9933
	Ceftolozane	−81.014	−68.1355	13.38447	−12.4592	62.71666	−52.3695
	Meropenem	−56.7565	−19.4327	2.120 725	−0.34663	33.59121	−43.3942

### Per residue binding free energy decomposition analysis

The decomposition of the binding energy into 5 key mutated residues is shown in this analysis. These mutated residues are closely connected with residues involved in the hydrolytic reaction β-lactam antibiotics and show altered binding with meropenem. This results in alterations in the active site structure and binding with β-lactam antibiotics and hence results in varied energy contribution with meropenem. Met69Cys, Glu104Tyr, Tyr105His, Ala237Ser, and Gly238Cys mutations result in increasing the dG bind energy in SME-1, as compared to that in TEM-1 with the complex with meropenem. As such, these residue mutations must be responsible for increasing the affinity of meropenem for SME-1, compared to TEM-1.

## Discussion

RMSD, PCA, and MMGBSA of the MD trajectory show the difference in the binding affinity of SME-1, as compared to wild-type TEM-1 in complex with meropenem (Fig. 3, 7, and Table 5).

Key active site residues, which are of catalytic importance Ser70, Lys73, Ser130, Asn132, Glu166, and Asn170 play a critical role in modulating the substrate activity. They are usually conserved but could be relocated due to structural modification of the enzyme. These relocations could be caused by a mutation in other residues close to the active site, which plays an important role in the structural plasticity of the enzyme. Further, the structure and activity relationship of these enzymes have been discussed for giving insights into the mechanism of action of SME-1 enzymes, for becoming antibiotic resistant.

Multiple sequence alignment reveals the presence of some unique mutations in SME-1, as compared to other naturally mutated β-lactamase enzymes considered in this study and wild-type TEM-1 enzymes (Table 1). Among these, Met69Cys, Glu104Tyr, Tyr105His, Ala237Ser, and Gly238Cys are closely connected with residues such as Ser70, Lys73, Ser130, Asn132, Glu166, and Asn170, which participate in the hydrolytic reaction with meropenem (Fig. 8).

It can be observed from Fig. 1d that Ser70 of SME-1 is displaced, as compared to that of SHV-1 and TEM-1. This observation is derived from the PDB structure not complex with meropenem. However, we further studied SME-1, SHV-1, and TEM-1 in the complex with meropenem at 50 ns of MD simulation (most stable state). Fig. 10, S2, S3, S4,†Table 2, and Table 4 depict the deviation of Ser70 residue along with residues that are involved in the hydrolytic reactions in SME-1, as compared to that in TEM-1 and SHV-1 in complexation with meropenem at 50 ns of MD simulation (stable state). Table 2 shows the RMSD value of Ser70 residue 0.071 in SME-1, as compared to TEM-1. Further, Table 3 shows the RMSF value of the Ser70 residue to be 0.755 in SME-1, which is greater, compared with that in TEM-1 and SHV-1.

Table 2 shows the deviation of Ω-loop (residue 164–179), SDN loop (residue 130–132), and other residues, which are involved in the hydrolytic reaction with β-lactam antibiotics in SME-1, as compared to other carbapenemase considered in this study with respect to wild type TEM-1. Further, SME-1 in the complex with meropenem shows the maximum deviation in conserved residues like Ser70, Lys73, Ser130, Asn132, Glu166, and Asn170 compared to naturally mutated SHV-1 and wild-type TEM-1 class A β-lactamase enzymes (Table 4 and Fig. 10).

Residues 69, 70, 73, 104, 105, 166, 170, 237, and 238 (mutated residues and residues, which are involved in the hydrolytic reaction with β-lactam antibiotics) showed a significant deviation in naturally mutated class A β-lactamase enzyme SME-1, as compared to naturally mutated class A β-lactamase enzyme SHV-1 and wild class A β-lactamase enzyme TEM-1. Ω-loop forms the wall of the active site and is the most flexible element of the active site. Hence, its structure alteration depicts an altered active site structure of SME-1 enzyme, as compared to TEM-1 in complex with meropenem (Fig. 10b).

**Fig. 9:**
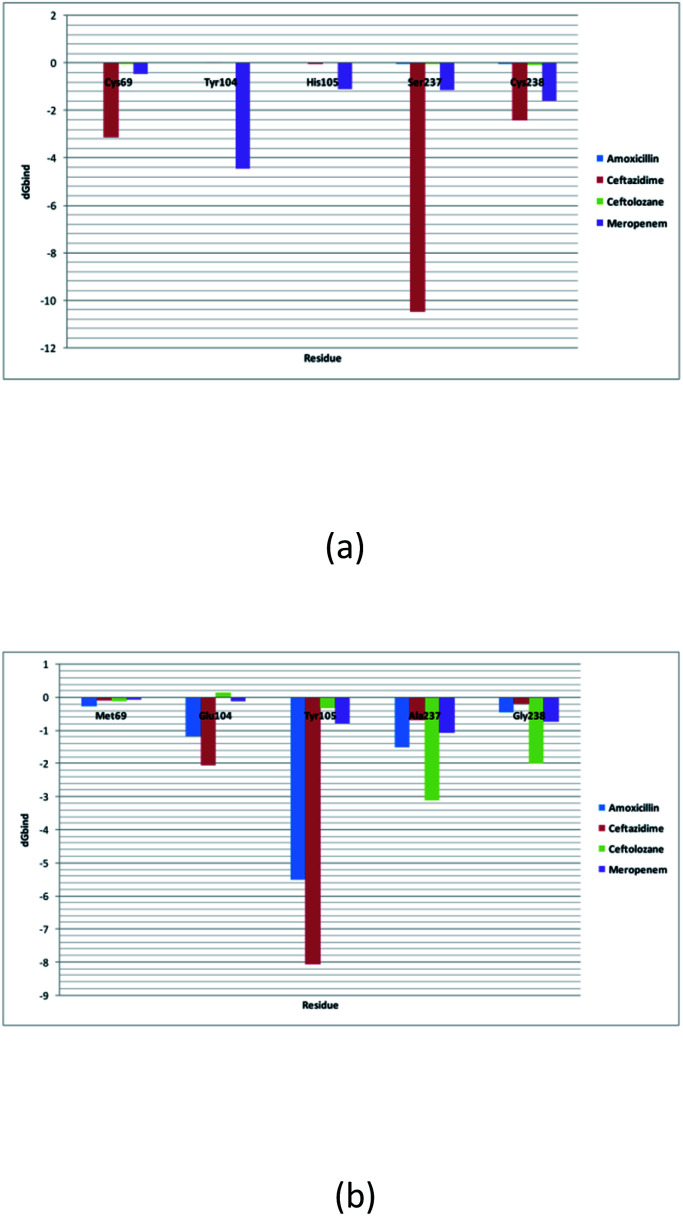
(a) Shows the residue decomposition analysis for (a) clinically mutated class A β-lactamase enzyme SME-1 and (b) wild class A β-lactamase enzyme TEM-1.

**Fig. 10:**
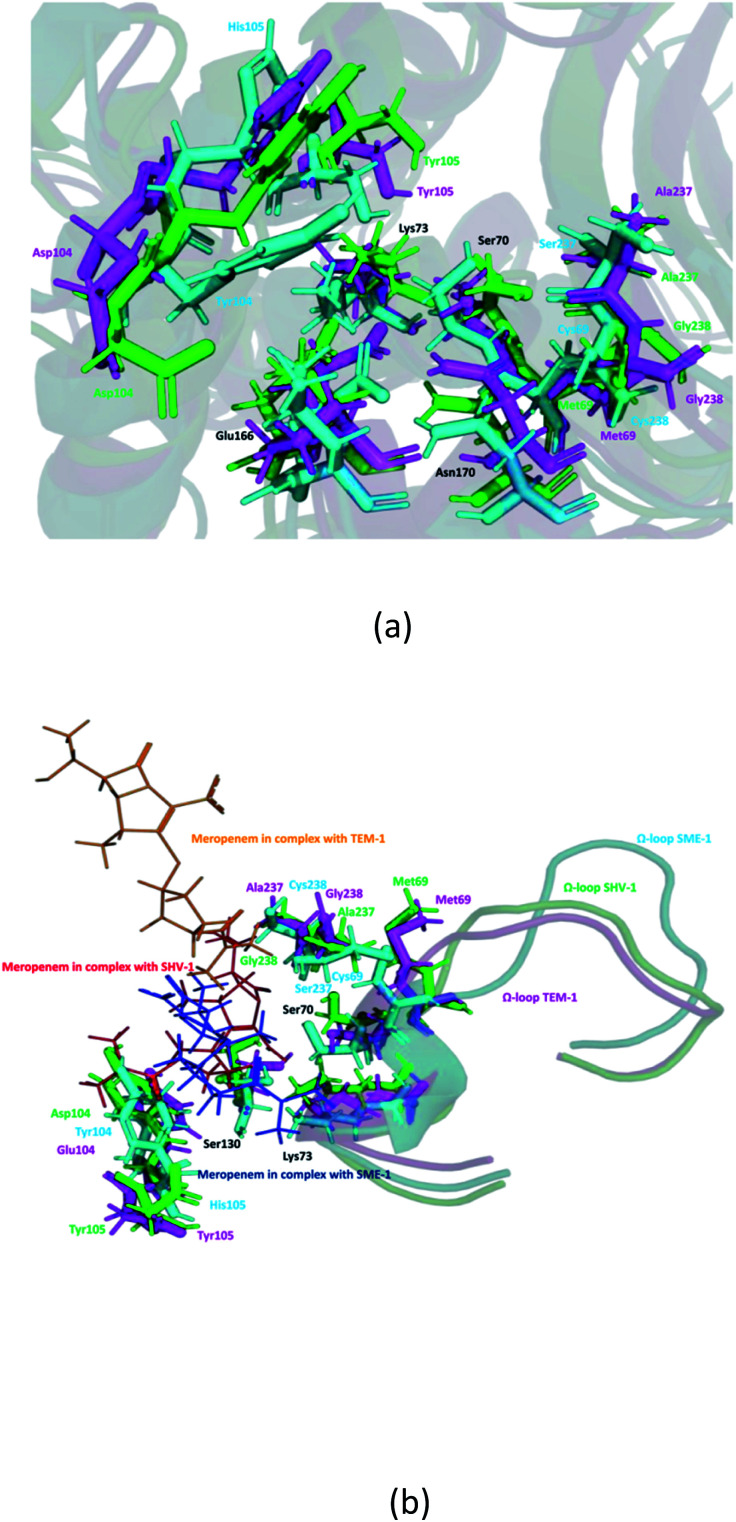
(a) Shows residues that are involved in the hydrolytic reaction with β-lactam antibiotics and are conserved (black), mutated in SME-1 (cyan), SHV-1 (green) and TEM-1 (magenta), at 50 ns (stable state of MD simulation) (b) Shows modifications in Ω-loop structure at 50 ns (stable state of MD simulation).

Alterations in the H-bonding pattern can be observed in Tyr104 of SME-1, as compared to those in Asp104 of SHV-1 and Glu104 of TEM-1 (Fig. 2d, h, and l). Tyr104 has an increased interaction fraction in SME-1, as compared with that in Asp104 in SHV-1 and Glu104 in TEM-1 with meropenem (Fig. 5d, h and l). Tyr104 is directly linked with Leu167 residue, which is further linked to Asn170 and Asn132 residues that are important catalytic residues in SME-1 (Fig. 8a). Whereas, Glu104 is linked with the Pro167 residue, which is further linked with only Asn170 in wild-type TEM-1 enzyme (Fig. 8b). Further, it can be observed that Tyr104 has a lower dG binding in SME-1, as compared to that in Glu104 in TEM-1 in complex with meropenem (Fig. 9). As 104 residue is closely connected with residues, which are involved in the hydrolytic reaction with β-lactam antibiotics such as Asn130 and Asn170, mutation results in the displacement of Tyr104 in SME-1 with respect to Asp104 in SHV-1 and Glu104 in TEM-1 with meropenem, as shown in the 50 ns time frame of the MD simulation (Fig. 10). So, it can be concluded that Glu104Tyr mutation results in some structural modifications in the active site of SME-1 and as it is linked to residues, which are involved in the hydrolytic reaction with β-lactam antibiotics such as Asn170 (part of Ω-loop) and Asn132 (part of SDN loop), as compared to wild type TEM-1.

His105 of SME-1 forms a polar interaction with meropenem, whereas Tyr105 of SHV-1 and TEM-1 forms a hydrophobic interaction with meropenem (Fig. 2d, h, and l). His105 in SME-1 forms a link with Ser130, Asn132 and Tyr129 (Fig. 8a). Tyr105 in TEM-1 forms a link with Ser130 and Asn132 (Fig. 8b). Both the residues are of catalytic importance and form part of the SDN loop. His105 in SME-1 has a lower dG binding value, as compared to Tyr105 in TEM-1 (Fig. 9). His105 in SME-1 shows a structural deviation, as compared to Tyr105 of SHV-1 and TEM-1 (Fig. 10), and is linked to residues, which are involved in the hydrolytic reaction with β-lactam antibiotics and loops like Ser130, Asn132, and SDN loop, respectively. This observation was made in a complex with meropenem at 50 ns of MD simulation (stable state). So, it can be concluded that the Tyr105His mutation results in structural modifications that could alter the binding of ligands such as meropenem.

Similarly, Ala237Ser and Gly238Cys mutations in SME-1 result in H-bond formation in SME-1, which is absent in SHV-1 and TEM-1 with meropenem. A previous study demonstrated that the formation of a disulphide bond in class A β-lactamse enzymes at Cys69 and Cys238 is important for the catalytic activity against most of the β-lactam antibiotics including carbapenems.^16^ Ser237 and Cys238 are directly linked with Cys69 in SME-1 (Fig. 8a). These interactions are altered in TEM-1, as the Met69 residue is connected with Ala237 and Ser234, which is further connected with Gly238 (Fig. 8b).

Active site structure is directly related to the function and activity of an enzyme. Ω-Loop structure alteration in the case of SME1 (compared to wild TEM1 and clinically mutated SHV1) shows its altered active site at 50 ns, which is a stable state of molecular dynamic simulation. It can be observed from the residue decomposition analysis that the dGbind value is different in the case of all the key mutated residues such as 69, 104, 105, 237, and 238 in SME1, as compared with that in TEM1. Further, it can be observed from Fig. 10 that the mutated (Met69Cys, Glu104Tyr, Tyr105His, Ala237Ser, and Gly238Cys) and active site catalytically important residues (Ser70, Lys73, Ser130, Asn132, Glu166, and Asn170) are relocated in SME1, as compared with TEM1 and SHV1. These observations give evidence of the structural analysis, which is correlated with the enzymatic activity with residues.

As residues 69, 104, 105, 237, and 238 are closely connected to residues, which are involved in the hydrolytic reaction with β-lactam, antibiotics such as Ser70, Lys73, Ser130, Asn132, Glu166, and Asn170, and their mutation results in altered interactions with residues, which are involved in the hydrolytic reaction with β-lactam antibiotics, which are involved in the catalytic reaction with meropenem as well as with meropenem, which is a carbapenem. These structural modifications could result in active site plasticity and altered binding with carbapenems in SME-1 class A β-lactamase enzymes. Hence, it could be concluded that Met69Cys, Glu104Tyr, Tyr105His, Ala237Ser, and Gly238Cys mutations occur in SME-1 and result in altered interactions with residues, which are involved in the hydrolytic reaction with β-lactam antibiotics.

## Materials and methods

### Kinetics analysis

The catalytic efficiency (*K*_cat_/*k*_m_) was derived based on a literature survey for the kinetic analysis of SME-1 with other naturally mutated and wild-type classes A β-lactamase enzymes.

### Sequence analysis

FASTA sequences of 14 naturally mutated class A β-lactamase enzymes TEM-52 (PDB ID: 1HTZ), SHV-1 (PDB ID: 1SHV), SME-1 (PDB ID: 1DY6), NMC-A (PDB ID: 1BUE), SED-1 (PDB ID: 3BFE), CTXM-9 (PDB ID: 1YLJ), CTXM-27 (PDB ID: 1YLP), PENI (PDB ID: 3W4P), GES-2 (PDB ID: 3NI9), GES-11 (PDB ID: 3V3R), GES5 (PDB ID: 4GNU), PER-1 (PDB ID: 1E25) and PER-2 (PDB ID: 4D2O) and wild type class A β-lactamase enzymes (TEM-1) were derived from RCSB-PDB database. These sequences were compared using the multiple sequence alignment tool Multalign.^17^ Furthermore, a phylogenetic tree was formed using Clustal W.

### Structure analysis

The structure of 14 naturally mutated class A β-lactamase enzymes TEM-52 (PDB ID: 1HTZ), SHV-1 (PDB ID: 1SHV), SME-1 (PDB ID: 1DY6), NMC-A (PDB ID: 1BUE), SED-1 (PDB ID: 3BFE), CTXM-9 (PDB ID: 1YLJ), CTXM-27 (PDB ID: 1YLP), PENI (PDB ID: 3W4P), GES-2 (PDB ID: 3NI9), GES-11 (PDB ID: 3V3R), GES5 (PDB ID: 4GNU), PER-1 (PDB ID: 1E25) and PER-2 (PDB ID: 4D2O) were derived in the PDB format from RCSB-PDB database. PyMOL Molecular Graphics System, Version 2.0 Schrodinger, LLC (academic version) was used to visualize and compare enzyme structure of naturally mutated and wild-type class A β-lactamase enzymes. Furthermore, RMSD values of all the naturally mutated class A β-lactamase enzymes were calculated, as compared to wild-type TEM-1 using the PyMOL Molecular Graphics System, Version 2.0 Schrodinger, LLC (academic version). Structures of SME-1, SHV-1, and TEM-1 in complex with meropenem at 50 ns (stable state during MD simulation), were aligned to each other for comparing their structural features.

### Molecular docking and MMGBSA

Protein preparation wizard suite v10.2, Schrodinger, LLC, New York, NY, 2015, was used for the protein preparation of 14 naturally mutated class A β-lactamase enzymes and wild-type TEM-1. Default parameters were used for scraping off steric clashes, non-essential water molecules, and hetero-atoms.^18^ The dimensions grid box created around the active site of the target receptor were kept as inner box: *X* = 20, *Y* = 20, *Z* = 20, outer box: *X* = 20, *Y* = 20, *Z* = 20 and was centered around active site residues 70, 73, 130, 132, 166, 170 and 234 using Glide v6.6, Schrodinger, LLC, New York, 2015 module.^19^ Ligand processing was performed using Ligprep along with the Epik module and expansion of protonation and tautomeric states (7.0 + 2.0 Ph units). Five stereoisomers of low energy conformer were generated for each ligand, out of which a single three-dimensional structure (lowest energy conformation) with accurate chirality was selected for further investigation. Molecular docking of 14 naturally mutated β-lactamase enzymes, as well as a wild-type class A β-lactamase TEM-1, with amoxicillin (PubChemID: 33 613), ceftazidime (PubChemID: 5 481 173), ceftolozane (PubChemID: 56 843 746) and Meropenem (PubChemID: 441 130) using GLIDE v6.7 (Schrodinger, LLC, New York, 2015). Amoxicillin is penicillin, ceftazidime and ceftolozane are cephalosporins, and meropenem is a carbapenem.

Molecular mechanics/generalized Born surface area (MM–GBSA) of the docked protein-ligand complex was performed using Prime, Schrodinger, LLC, New York, NY, 2019–2, to evaluate the binding affinity between the enzyme and the ligand.

### Molecular dynamic simulation

Molecular dynamic (MD) simulation was executed using Desmond, incorporated in the Schrodinger suite. The OPLS 2005 force field was used for SME-1, SHV-1, and wild-type class A β-lactamase TEM-1. The TIP3P water model was enclosed in an orthorhombic box (*a* = 10 Å, *b* = 10 Å, *c* = 10 Å) along with periodic boundary conditions. A salt concentration of 0.15 mol L^−1^ was used for charge neutralization using cation (Na^+^) or anion (Cl^−^). Energy minimization was performed using the steepest descent method with a convergence threshold of 1.0 kcal mol^−1^ Å^−1^. The system's minimization and relaxation were performed using an NPT ensemble, and a 300 K temperature was maintained along with 1.013 bar pressure. MD simulation was performed for 100 ns. MD trajectory analysis was carried out *via* RMSD, RMSF, and hydrogen bond analysis.^20^

### MMGBSA of the MD trajectory

MMGBSA calculations of all protein-ligand complex MD trajectories were performed using a prime package of Schrödinger LLC. MMGBSA can be mathematically defined as follows:*G* = *E*_bnd (BONDED ENERGY)_ + *E*_ele (ELECTROSTATIC ENERGY)_ + *E*_vdw (VAN DER WAALS ENERGY)_ + *G*_solv (NON-POLAR SOLVATION ENERGY)_ + *G*_npr (SOLVENT ACCESSIBLE SURFACE AREA ENERGY)_ − TS^21^

### Principle component analysis

Principle component analysis (PCA) of molecular dynamics simulation helps in understanding the essential dynamics of the protein complex. Desmond simulation package of Schrödinger LLC was used for finding the essential dynamics of the 12 complex (including SME-1, SHV-1, and TEM-1 protein in complex with amoxicillin, ceftazidime, ceftolozane, and meropenem).

### Residue interaction analysis (RIN)

RIN demonstrates inter-residue interactions with the help of network formations. This method gives insights into the effect of mutation on drug resistance by showing how the interactions are altered in the case of mutated residues.^22^ Protein (SME-1 and TEM-1) residue interaction networks were downloaded from the RING web server.^23^ RIN analysis was performed using Cytoscape.^24^

### Per residue binding free energy decomposition analysis

Binding free energy calculation gives insights into the energetic contribution of the protein, binding to a ligand. This helps in understanding the specificity and sensitivity of protein-ligand interactions. This was calculated using a prime package of Schrödinger LLC. The decomposition of binding energy per residue helps in knowing the energy of different residues, which could be important for altering the binding pattern of the protein-ligand complex.^25^

## Conclusions

SME-1 is a carbapenemase, which belongs to class A β-lactamase enzymes. Multiple sequence and structure alignment shows the presence of some unique mutations, which results in the altered binding of SME-1, as compared to other naturally mutated class A β-lactamase enzymes and wild-type TEM-1. SME-1 has altered binding with β-lactam antibiotics as evident from the molecular docking, molecular dynamic simulation, PCA, and MMGBSA analysis of the MD trajectory of SME-1, SHV-1, and TEM-1 class A β-lactamase enzymes in complex with β-lactam antibiotics. Active site alterations occur in SME-1, which is evident from the altered Ω-loop structure and relocation of residues such as Ser70, which are involved in the hydrolytic reaction with β-lactam antibiotics. Molecular docking and molecular dynamic simulation interaction analysis showed that 69, 104, 105, 237, and 238 residues are mutated and show altered interaction with meropenem. Furthermore, these residues are closely connected with residues Ser70, Lys73, Ser130, Asn132, Glu166, and Asn170, which are involved in the hydrolytic reaction with β-lactam antibiotics including meropenem. Based on the altered interactions of 69, 104, 105, 237, and 238 residues with meropenem in SME-1, as compared with TEM-1 and its close connections with residues involved in the hydrolytic reaction, it can be concluded that Met69Cys, Glu104Tyr, Tyr105His, Ala237Ser, and Gly238Cys mutations result in structural and binding alterations with meropenem, leading to carbapenemase activity of the SME-1 class A β-lactamase enzyme.

## Conflicts of interest

There are no conflicts to declare.

## Supplementary Material

RA-012-D2RA02849B-s001
