# Supplementary material for: Mutations responsible for the carbapenemase activity of SME-1
Source: RSC Adv. 2022 Aug 15;12(35):22826–42. doi: 10.1039/d2ra02849b (PMC9377157; doi:10.1039/d2ra02849b)
Supplement: RA-012-D2RA02849B-s001 [file RA-012-D2RA02849B-s001.pdf]

Electronic Supplementary Material (ESI) for RSC Advances.
This journal is © The Royal Society of Chemistry 2022

Mutations responsible for the antibiotic resistance in SME-1

Vidhu Agarwal, Akhilesh Tiwari, Pritish Varadwaj*,

Indian Institute of Information Technology, Allahabad, India

**E-mail: pritish@iiita.ac.in, Tel: 9236666060, Full address: Indian Institute of Information Technology, Allahabad, Devghat, Jhalwa, Prayagraj-211015, U. P. INDIA*

S. No	Class A β-lactamase	β-lactam antibiotics	Catalytic efficiency K_{cat}/k_m ($\mu M^{-1} s^{-1}$)
2.	GES14	Benzylpenicillin	0.077
		Amoxicillin	0.054
		Ticarcillin	0.006
		Piperacillin	0.08
		Temocillin	0.00005
		Cephalothin	0.18
		Cephaloridine	0.034
		Nitrocefin	0.415
		Cefoxitin	0.0016
		Cefotaxime	0.0018
		Ceftazidime	0.000125
		Cefepime	0.0007
		Azetreonam	0.0007
		Imipenem	0.031
		Ertapenem	0.033
		Meropenem	0.005
3.	GES11	Benzylpenicillin	0.11
		Amoxicillin	0.19
		Ticarcillin	0.07
		Piperacillin	0.225
		Temocillin	0.00003
		Cephalothin	0.185
		Cephaloridine	0.13
		Nitrocefin	0.6
		Cefoxitin	0.0015
		Cefotaxime	0.29
		Ceftazidime	0.026
		Cefepime	0.01
		Azetreonam	0.017
		Imipenem	0.015

		Ertapenem	0.01
		Meropenem	0.035
5.	GES5	Benzylpenicillin	0.255
		Amoxicillin	0.26
		Ticarcillin	0.003
		Piperacillin	0.16
		Temocillin	0.0000007
		Cephalothin	0.1
		Cephaloridine	0.145
		Nitrocefin	0.470
		Cefoxitin	0.0005
		Cefotaxime	0.0014
		Ceftazidime	0.00014
		Cefepime	0.009
		Azetreonam	0.00003
		Imipenem	0.15
		Ertapenem	0.06
		Meropenem	0.06
8.	NMCA	Penicillin G	9.3
		Cephalothin	15.2
		Cefotaxime	3
		Ceftazidime	0.052
		Imipinem	11.3
		Ceftoxitin	0.062
		Azetronam	5.6
		Ticarcillin	0.07
9.	SME1	Penicillin G	0.8
		Ticarcillin	0.07
		Cephalothin	0.78
		Azetronam	0.28
		Imipinem	0.44
		Ceftoxitin	0.001
14.	CTXM9	Cephalothin	2
		Cefotaxime	3.7
		Ceftazidime	0.004
15.	CTXM27	Cephalothin	18
		Cefotaxime	9
		Ceftazidime	0.021
18.	SHV1	nitrocefin	14 ± 0.2
		ampicillin	18 ± 0.2
		piperacillin	16 ± 0.6
		cephalothin	0.9 ± 0.3
		cefotaxime	$\leq 0.001^{c}$
		ceftazidime	$\leq 2X10^{-5c}$
		cefepime	$\leq 0.004^{c}$

		Cephaloridine	0.22
		Cefoxitin	0.11
		Cefotaxime	0.024
		Ceftazidime	0.0017
		Cefepime	0.6
		Imipenem	0.081
19.	TEM1	Benzylpenicillin	84
		Nitrocefin	17
		Cephaloridine	2.2
		Cephalothin	0.65
		Cefotaxime	0.0015
		Ceftazidime	0.00007
		Cefoxitin	0.000006
		Imipenem	0.002
		Benzylpenicillin	0.000048
		Amoxycillin	0.000035
		Ticarcillin	0.000012
		Cephalothin	0.0000005

Table S1: Catalytic efficiency of clinically mutated class A β-lactamase enzymes with β-lactam antibiotics.

**Protein (class A β-lactamase)**	**Ligand**	**Term**	**Range**	**mean**	**median**	**Standard deviation**
SME-1 (1DY6)	Ceftazidime	RMSD_Backbone	[1.319,2.249]	1.805	1.806	0.150
		RMSD_Side_chain	[2.392,3.337]	2.799	2.770	0.169
		RMSD_Ligand	[0.000,1.043]	0.439	0.399	0.156
	Ceftolozane	RMSD_Backbone	[0.000,1.827]	1.254	1.264	0.183
		RMSD_Side_chain	[0.000,3.082]	2.292	2.287	0.283
		RMSD_Ligand	[0.000,3.233]	1.738	1.716	0.365
	Meropenem	RMSD_Backbone	[0.000,1.841]	1.110	1.476	0.236
		RMSD_Side_chain	[0.000,2.811]	2.306	2.379	0.268
		RMSD_Ligand	[0.000,2.607]	1.667	1.720	0.381
	Amoxicillin	RMSD_Backbone	[0.000, 1.653]	1.333	1.333	0.135
		RMSD_Side_chain	[0.000, 2.769]	2.430	2.474	0.172

		RMSD_Ligand	[0.000, 2.245]	1.765	1.666	0.423
SHV-1 (SHV1)	Ceftazidime	RMSD_Backbone	[0.000,2.13]	1.573	1.611	0.263
		RMSD_Side_chain	[0.000,3.07]	2.481	2.542	0.260
		RMSD_Ligand	[0.000,1.95]	1.275	1.305	0.234
	Ceftolozane	RMSD_Backbone	[0.000,1.957]	1.351	1.337	0.240
		RMSD_Side_chain	[0.000,2.930]	2.330	2.322	0.229
		RMSD_Ligand	[0.000,3.094]	2.105	2.495	0.757
	Meropenem	RMSD_Backbone	[0.000,1.994]	1.532	1.582	0.240
		RMSD_Side_chain	[0.000,3.356]	2.506	2.539	0.285
		RMSD_Ligand	[0.000,2.612]	1.431	1.523	0.613
	Amoxicillin	RMSD_Backbone	[0.000,2.06]	1.551	1.589	0.241
		RMSD_Side_chain	[0.000,3.36]	2.808	2.941	0.353
		RMSD_Ligand	[0.000,2.33]	1.224	1.139	0.395
TEM-1 (1ZG4)	Ceftazidime	RMSD_Backbone	[0.000,1.93]	1.226	1.158	0.261
		RMSD_Side_chain	[0.000,3.12]	2.264	2.180	0.357
		RMSD_Ligand	[0.000,2.12]	1.094	0.962	0.404
	Ceftolozane	RMSD_Backbone	[0.000,1.795]	1.132	1.133	0.152
		RMSD_Side_chain	[0.000,2.891]	2.237	2.272	0.192
		RMSD_Ligand	[0.000,3.627]	0.694	0.542	0.578
	Meropenem	RMSD_Backbone	[0.000,1.708]	1.283	1.330	0.199
		RMSD_Side_chain	[0.000,2.820]	2.200	2.236	0.241
		RMSD_Ligand	[0.000,2.679]	1.774	1.908	0.520
	Amoxicillin	RMSD_Backbone	[0.000,2.14]	1.526	1.528	0.250
		RMSD_Side_chain	[0.000,3.17]	2.403	2.400	0.285
		RMSD_Ligand	[0.000,2.84]	1.393	0.774	1.057

Table S2: RMSF mean, median mode values for all the class A β-lactamase and β-lactam antibiotic combination throughout 100ns molecular dynamic simulation.

**Protein PDB ID**	**Average B factor**	**Gamma atom B factor**	**Resolution (Å)**
1ZG4	22.106	23.83	1.55
4H8R	14.207	15.052	1.25
1SHV	22.106	23.83	1.98
2OV5	18.99	18.835	1.85
2QPN	15.651	16.413	1.10
1HTZ	46.134	46.439	2.40
1N9B	10.083	10.708	0.90
1E25	21.105	21.624	1.90
3NI9	25.897	26.23	2.00
1LHY	23.584	23.659	2.00
1LI0	23.387	23.742	1.61
1LI9	17.295	17.506	1.52

1YLJ	8.338	8.808	0.98
1IYS	9.382	9.543	1.65
1JWZ	16.957	17.351	1.80
1BTL	10.892	11.296	1.80
1YLP	12.005	12.95	1.20
1YLT	9.512	10.21	1.74
1YLW	11.199	11.462	1.74
4GNU	13.499	14.304	1.09
3DW0	10.473	10.635	1.60
4HBT	8.548	8.932	1.91
4D2O	27.371	27.435	2.20
1BUE	12.789	13.525	1.64
2GDN	19.22	19.53	1.72
3BLM	22.363	22.239	2.00
3TSG	22.178	22.05	1.90
1I2S	20.97	21.559	1.70
1W7F	26.823	26.758	1.80
1DY6	18.842	18.227	2.13
1HZO	9.741	10.133	1.75
2CC1	18.647	18.506	2.13
3BFE	17.942	17.789	2.40
3W4P	12.452	12.537	1.05
3W4Q	16.321	16.61	1.20
3V3R	20.09	20.057	1.90
1N4O	16.005	16.457	1.85
1O7E	10.992	11.156	1.51
4EQI	11.704	12.239	1.38

Table S3: Shows B-factor analysis results.

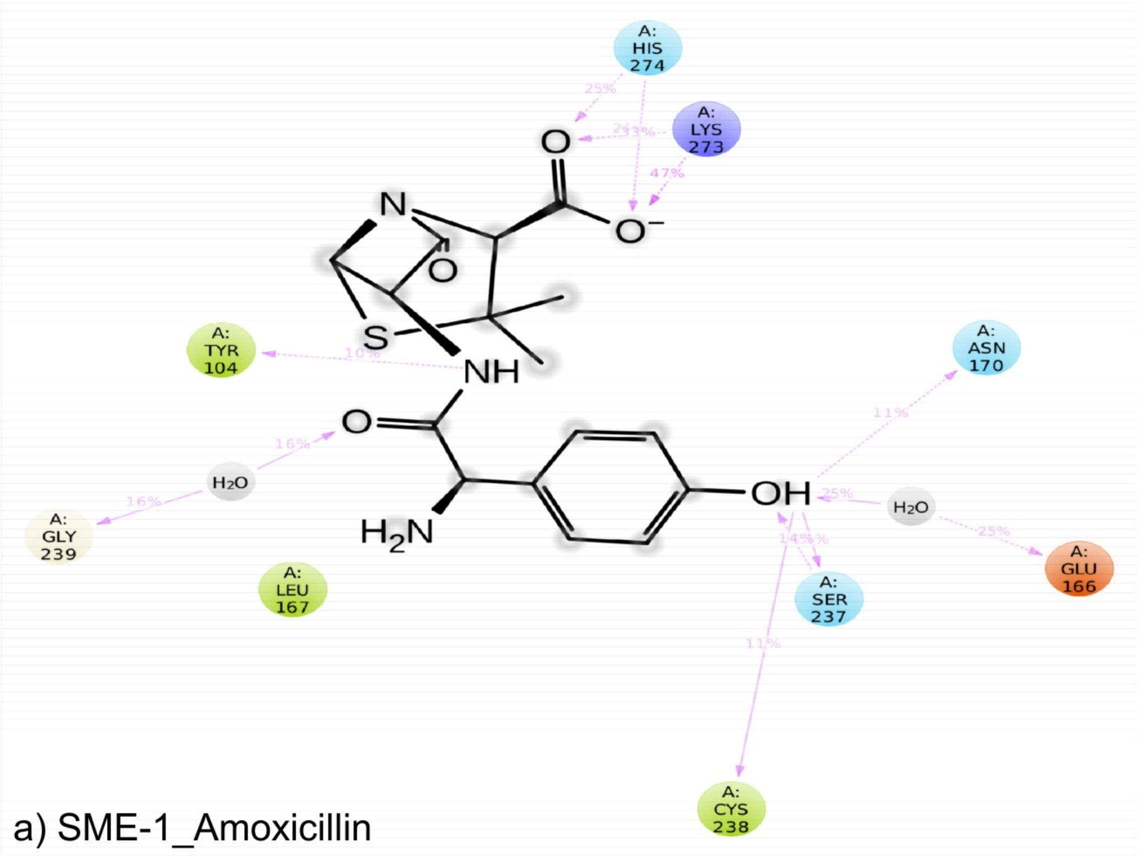

a) SME-1_Amoxicillin

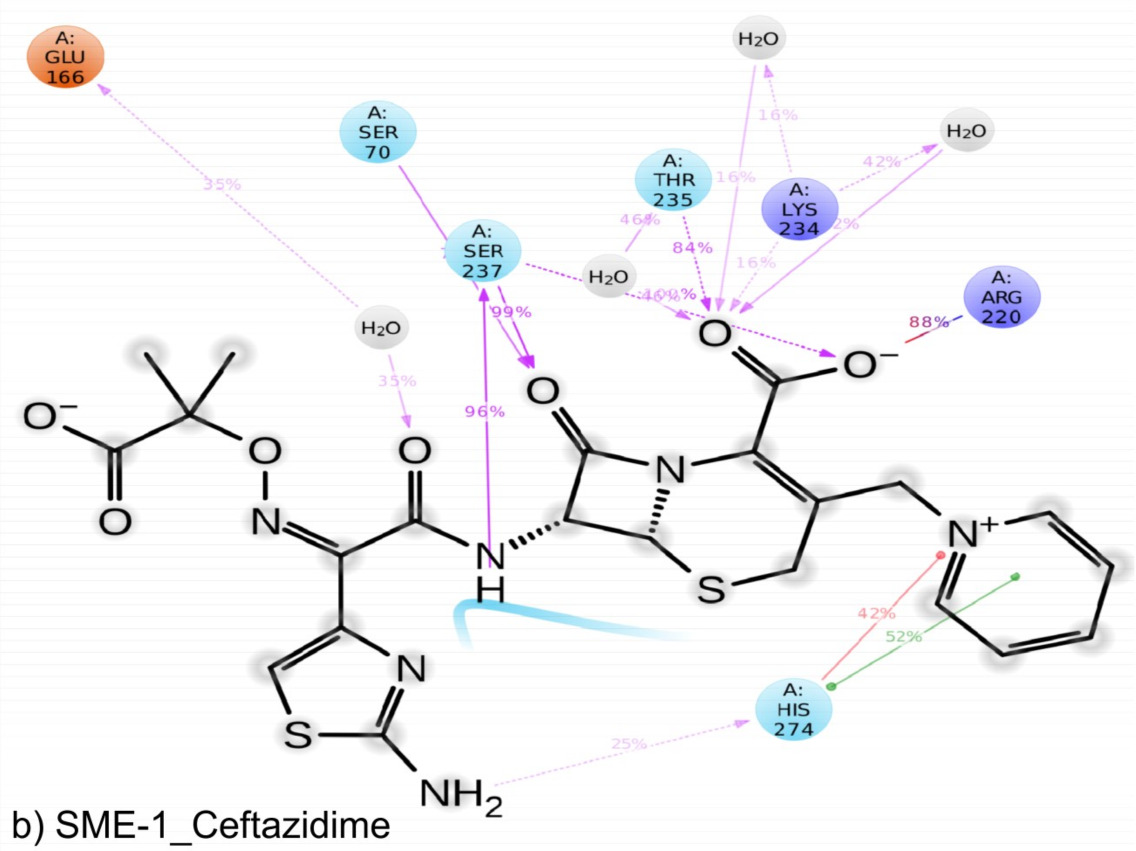

b) SME-1_Ceftazidime

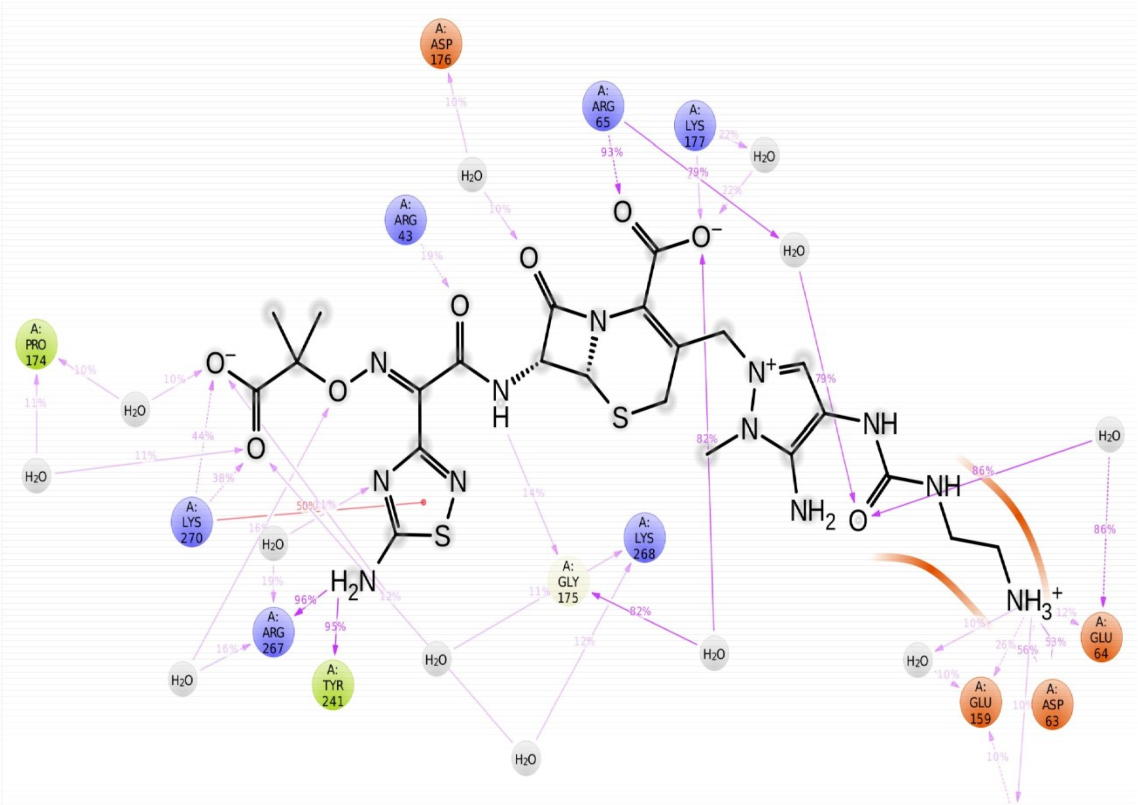

c) SME-1_Ceftolozane

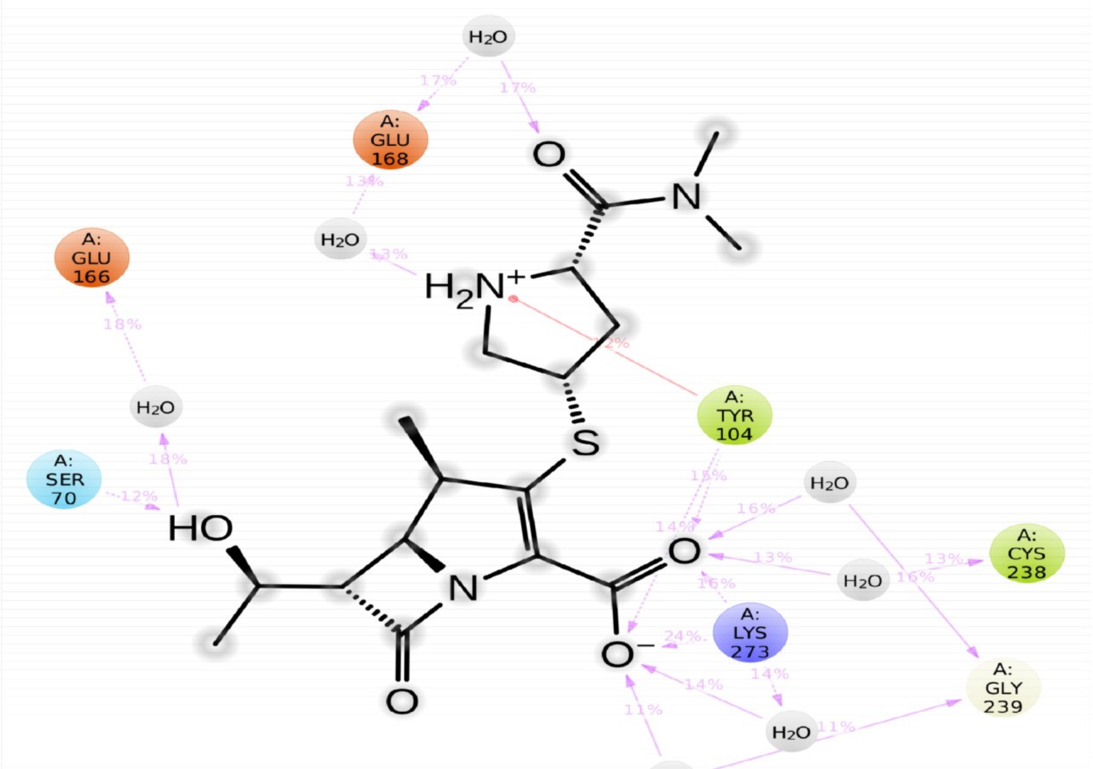

d) SME-1_Meropenem

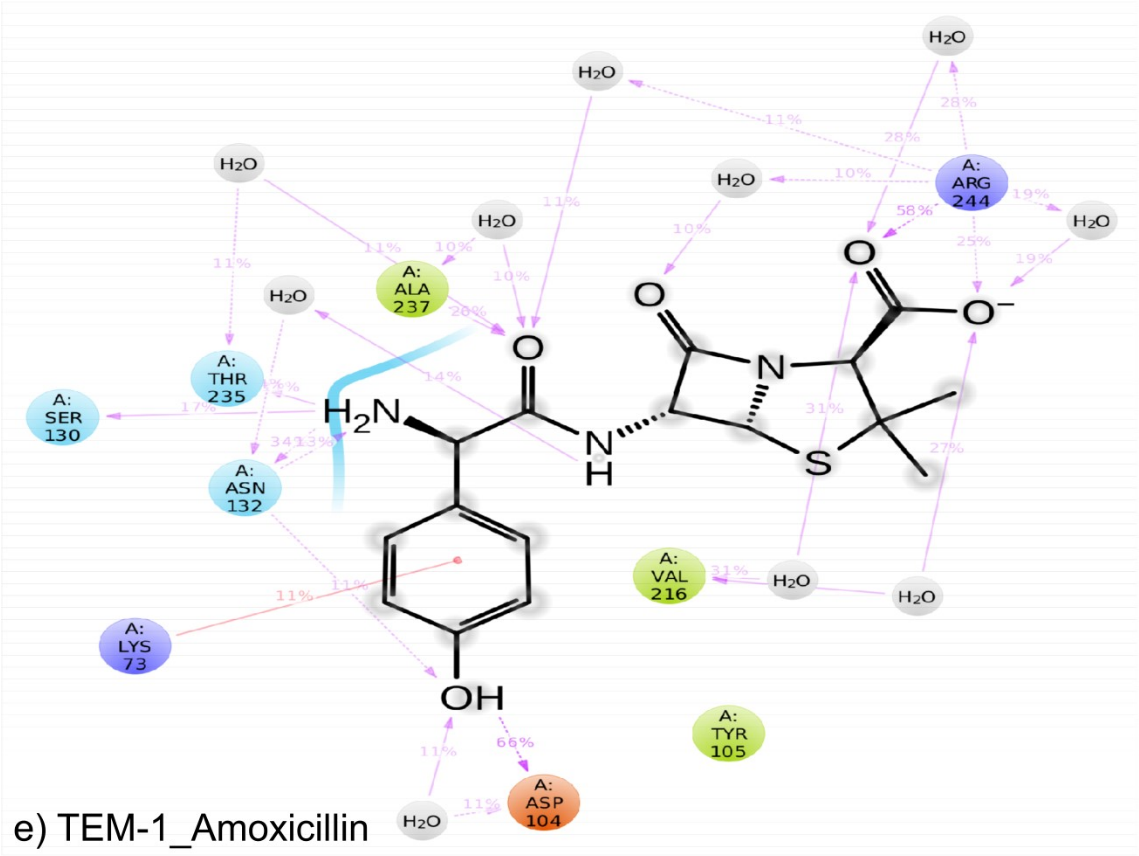

e) TEM-1_Amoxicillin

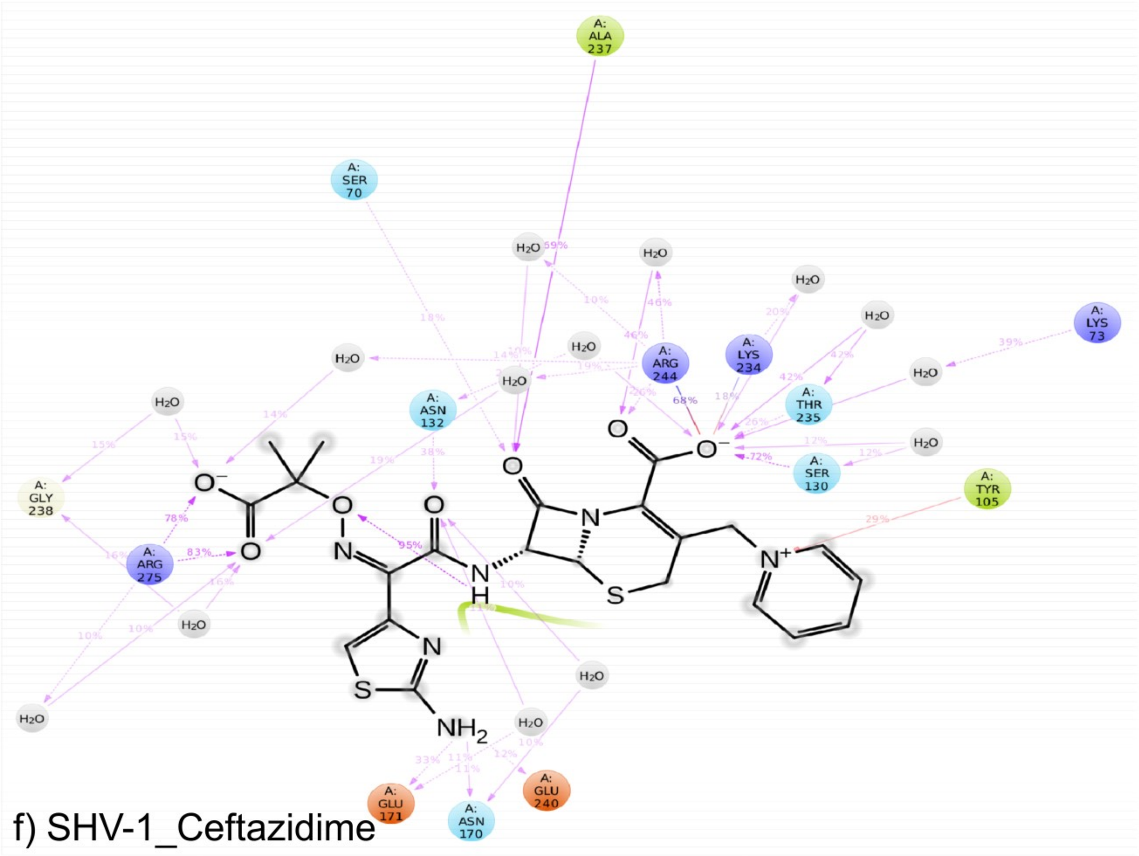

f) SHV-1_Ceftazidime

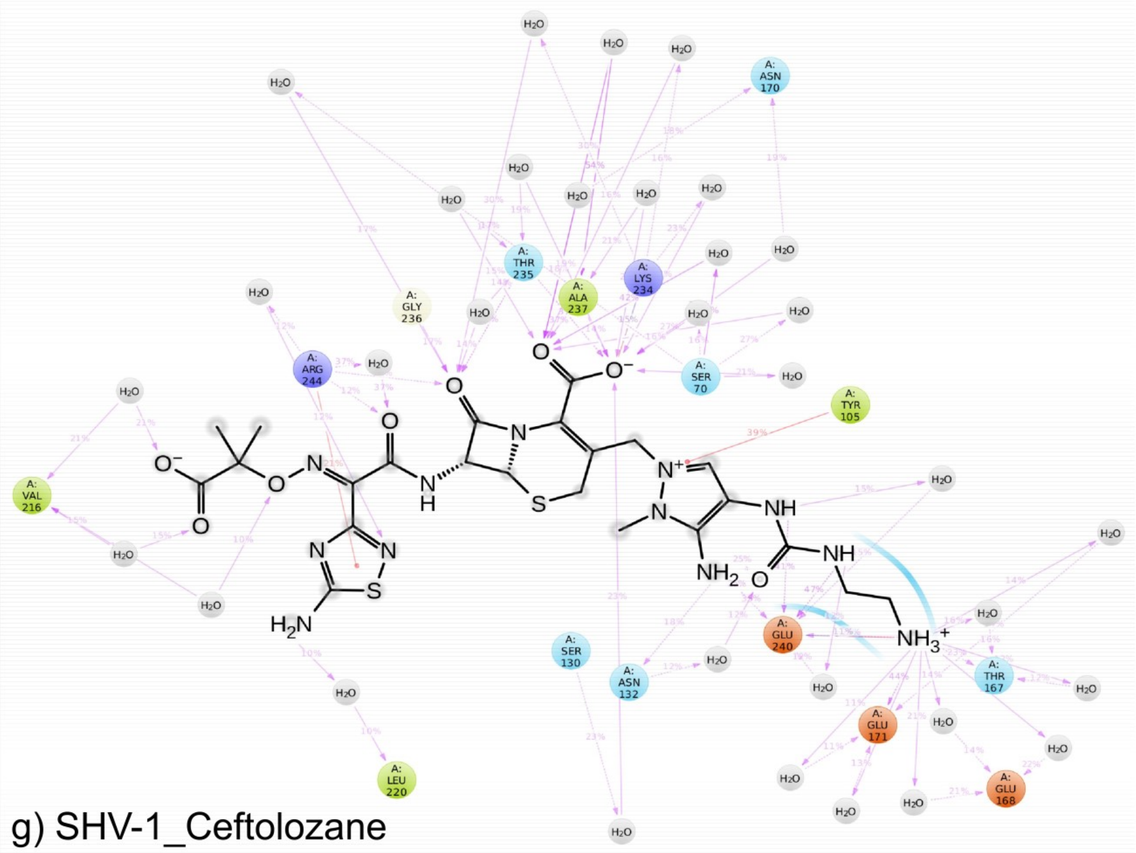

g) SHV-1_Ceftolozane

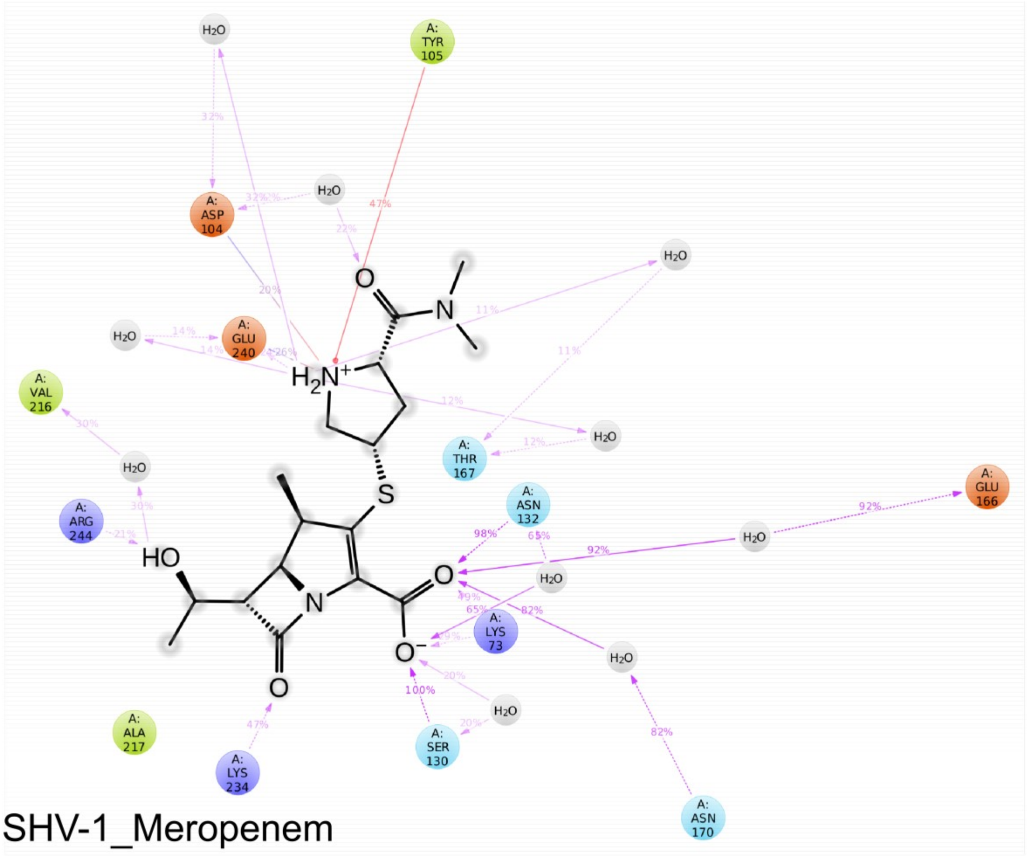

h) SHV-1_Meropenem

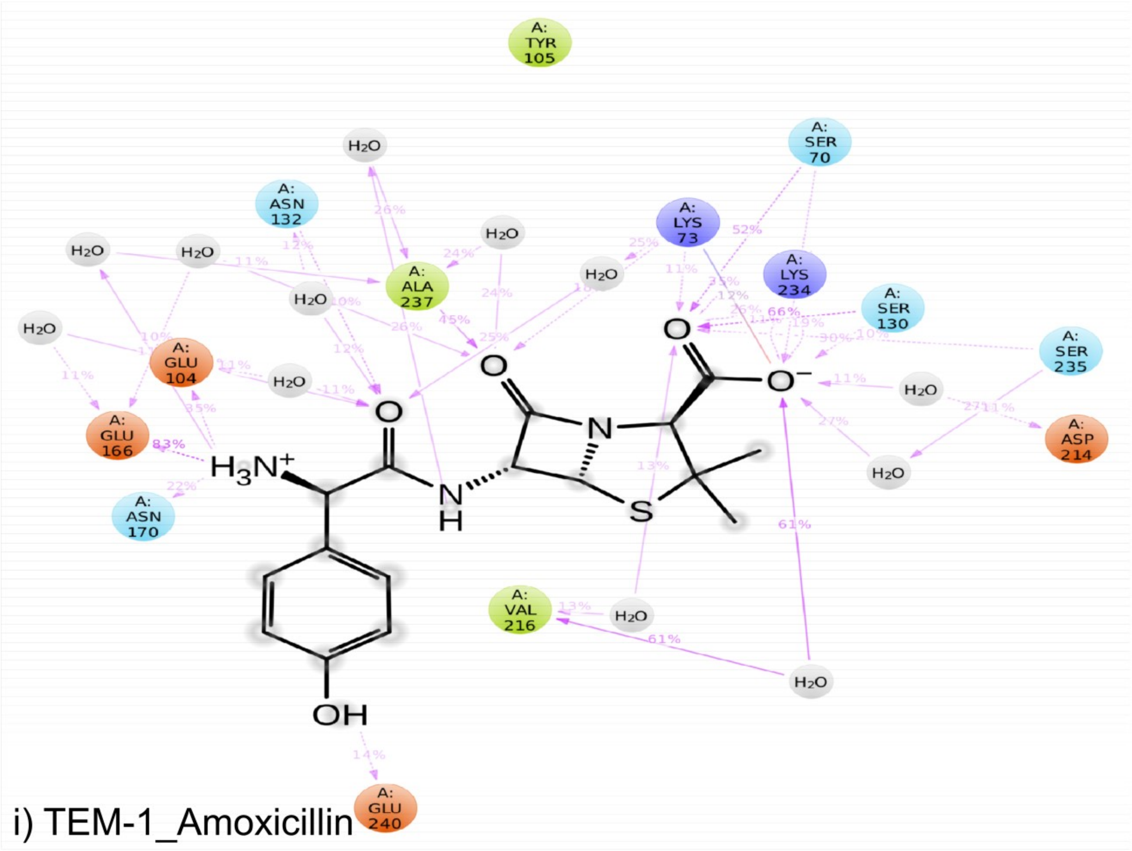

i) TEM-1_Amoxicillin

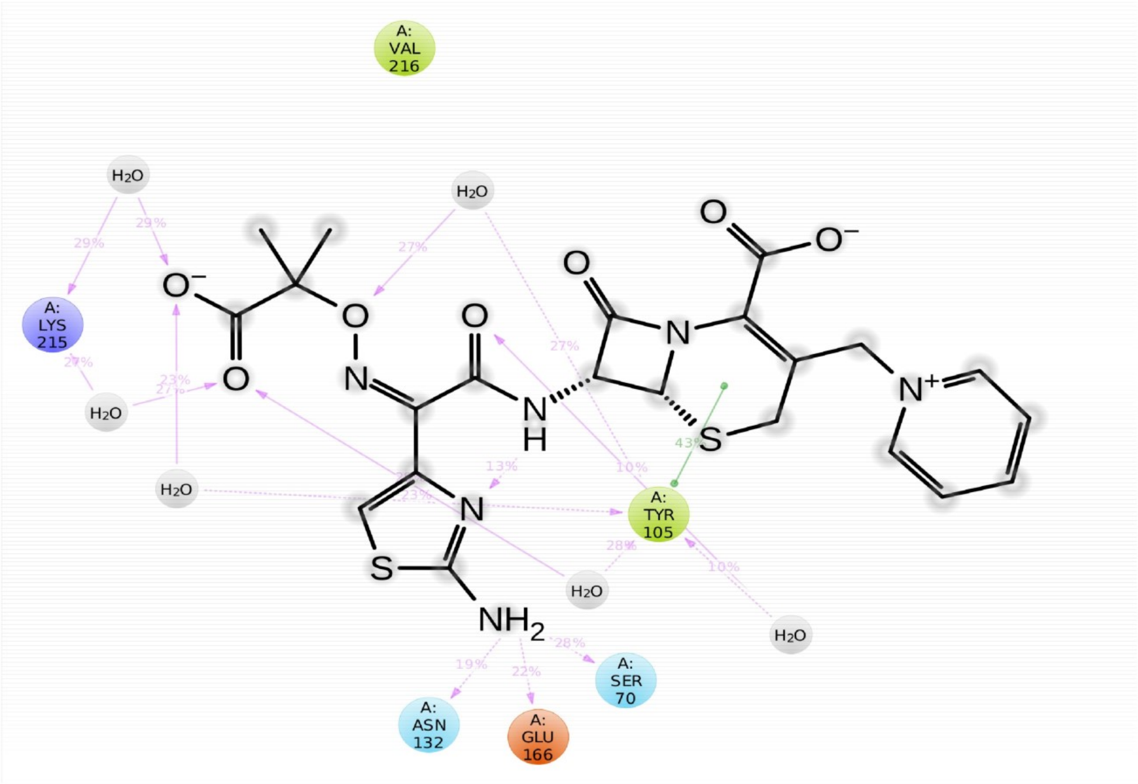

j) TEM-1_Ceftazidime

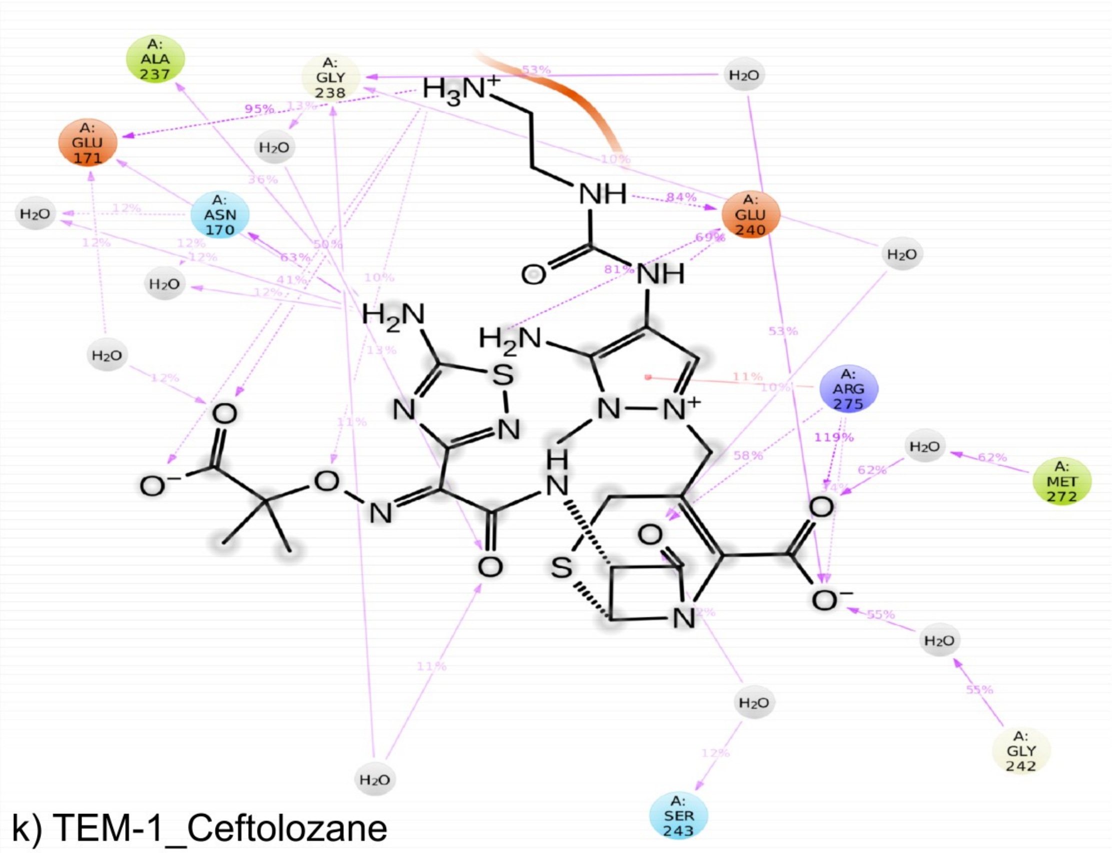

k) TEM-1_Ceftolozane

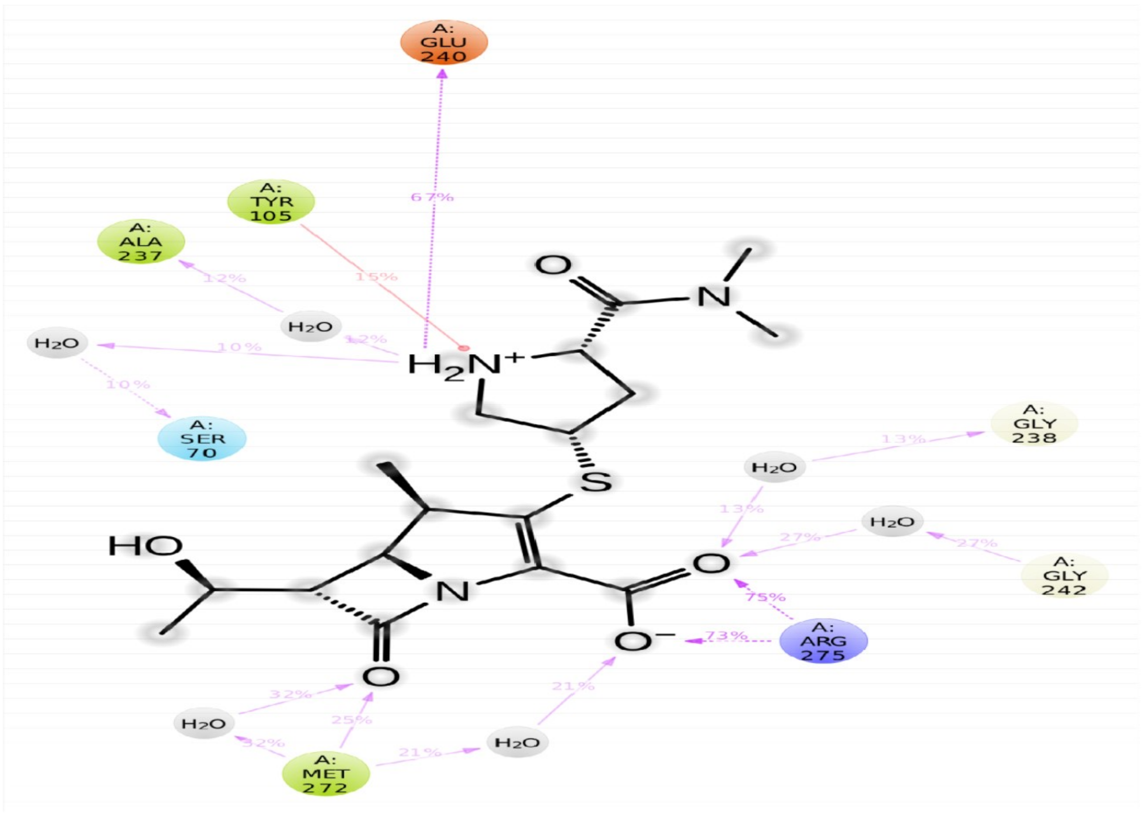

l) TEM-1_Meropenem

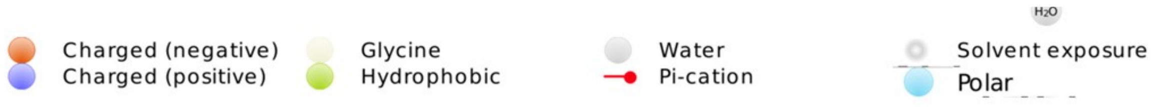

Figure S1: Shows the interaction diagram of the active site of (a) SME-1_Amoxicillin (b) SME-1_Ceftazidime (c) SME-1_Ceftolozane (d) SME-1_Meropenem (e) SHV-1_Amoxicillin (f) SHV-1_Ceftazidime (g) SHV-1_Ceftolozane (h) SHV-1_Meropenem (i) TEM-1_Amoxicillin (j) TEM-1_Ceftazidime (k) TEM-1_Ceftolozane (l) TEM-1_Meropenem.

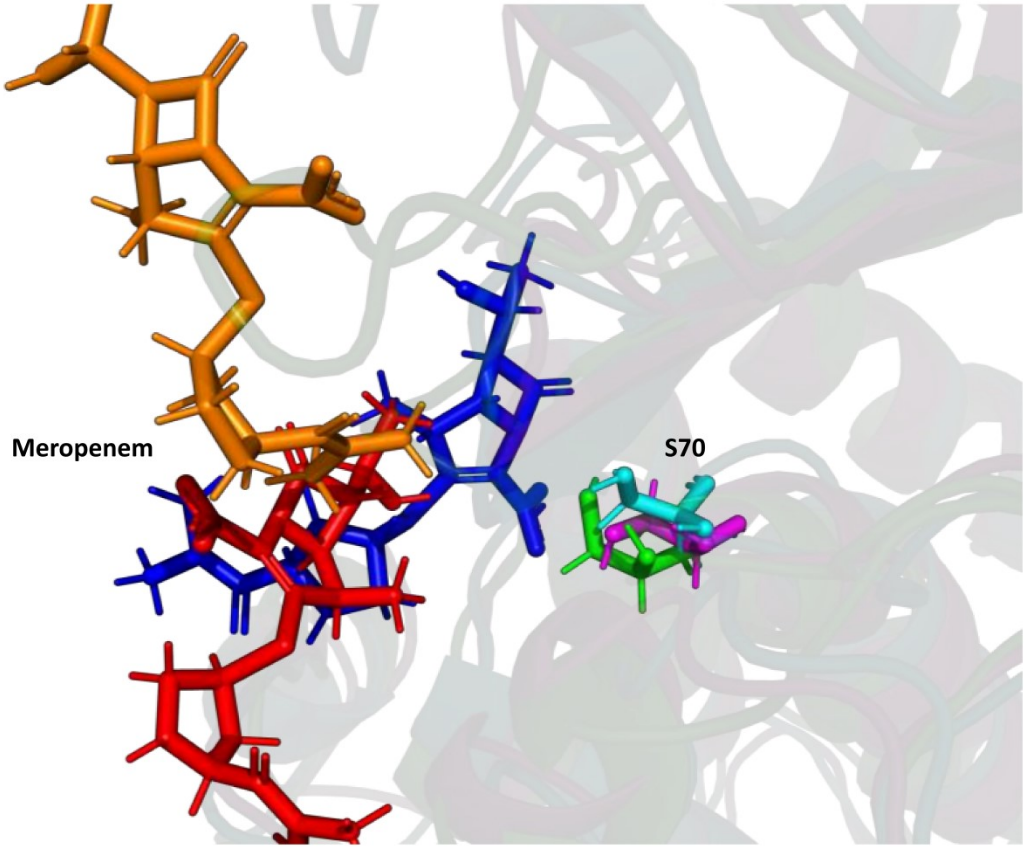

FigureS2: Shows the interaction between meropenem and S70 of SME-1 (green), SHV-1 (cyan) and TEM-1 (magentas), at 50 ns (stable state).

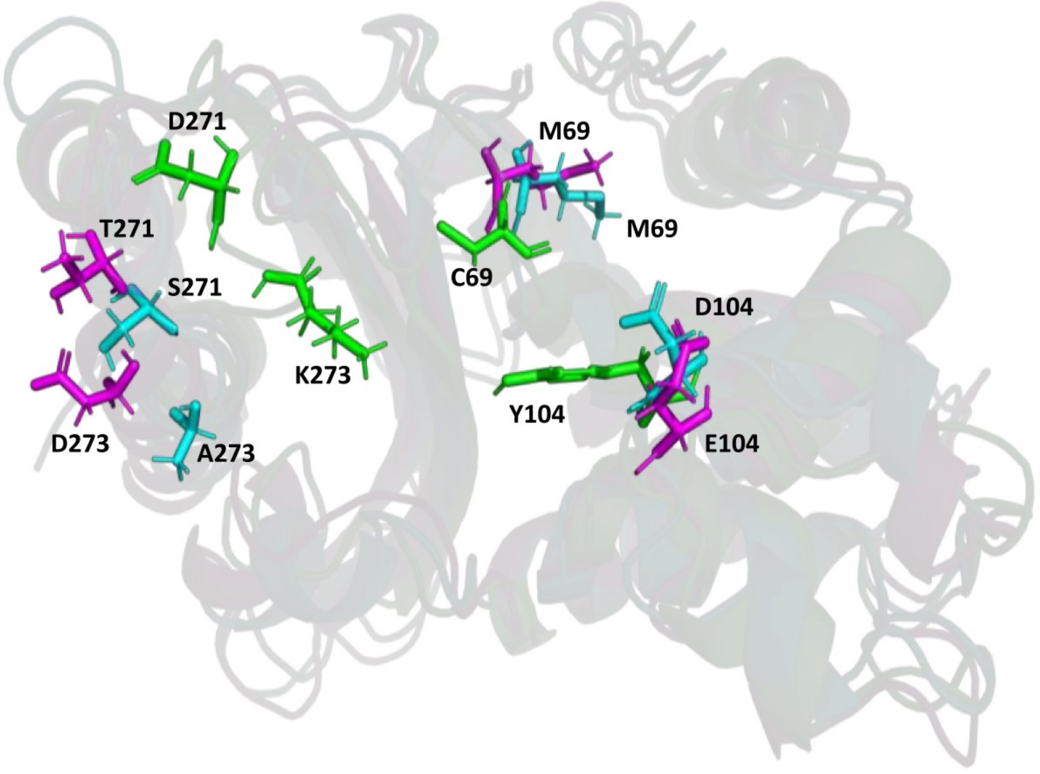

FigureS3: Shows catalytically important residues of SME-1 (green), SHV-1 (cyan) and TEM-1 (magentas) , at 50 ns (stable state).

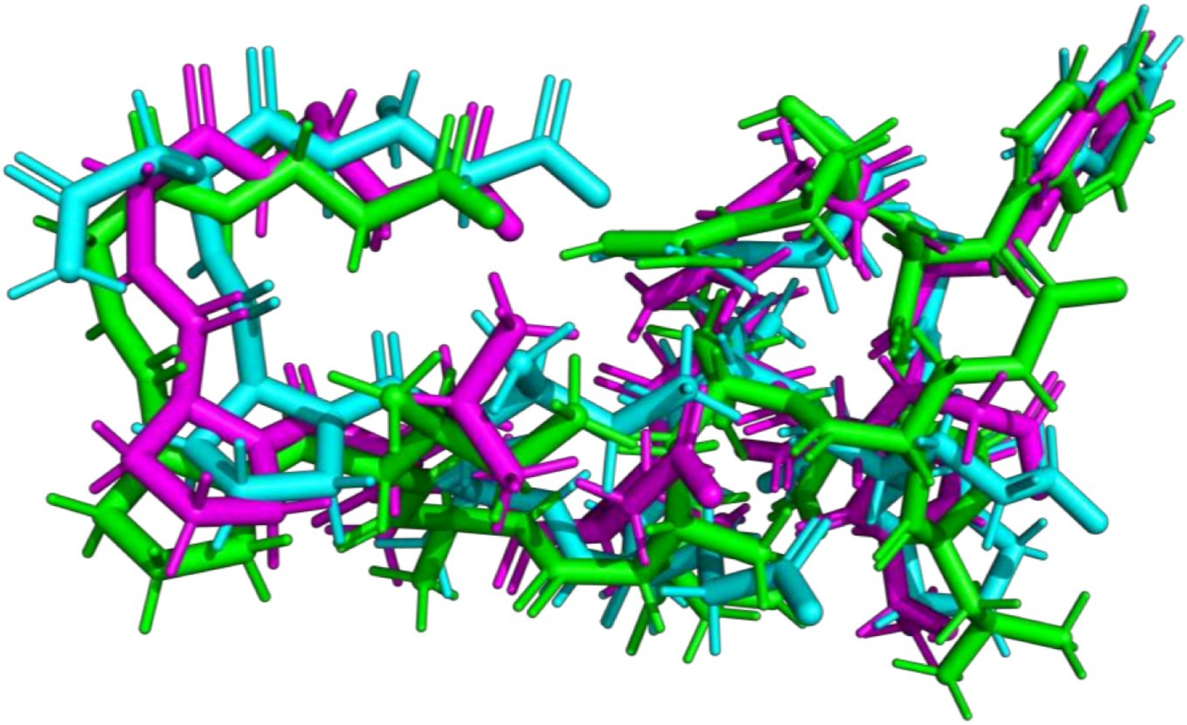

FigureS4: Ω-loop structure has been shown in stick model to compared SME-1 (green), SHV-1 (cyan) and TEM-1 (magentas) , at 50 ns (stable state).
